# Video transaction algorithm considering *FISCO* alliance chain and improved trusted computing

**DOI:** 10.7717/peerj-cs.594

**Published:** 2021-06-14

**Authors:** Yang Yang, Dingguo Yu, Chen Yang

**Affiliations:** 1 Intelligent Institute of Media, Communication University of Zhejiang, Hangzhou, China; 2 Finance Department, Hangzhou Vocational and Technical College, Hangzhou, China

**Keywords:** Alliance chain, Trusted computing, Video transaction, Video digest, Online transaction

## Abstract

With the advent of the era of self media, the demand for video trading is becoming more and more obvious. Alliance blockchain has the characteristics of traceable transaction records, tamper proof transaction records, decentralized transactions and faster transaction speed than public chains. These features make it a trading platform. Trusted computing can solve the problem of non Byzantine attack in the aspect of hardware. This paper proposes a video transaction algorithm considering FISCO alliance chain and improved trusted computing. First, an improved trusted computing algorithm is used to prepare a trusted transaction environment. Second, the video summary information extraction algorithm is used to extract the summary information that can uniquely identify the video. Finally, based on the video transactions algorithm of FISCO alliance chain, the video summary information is traded on the chain. Experimental results show that the proposed algorithm is efficient and robust for video transactions. At the same time, the algorithm has low computational power requirements and algorithm complexity, which can provide technical support for provincial and county financial media centers and relevant media departments.

## Introduction

2018–2020 were the most important years for the development of online video. In terms of video content provision, User Generated Content (UGC) has developed into Professional Generated Content (PGC)/ Occupationally-Generated Content (OGC), which will provide more professional content. Additionally, with the development of Multi-Channel Network (MCN), content that is easier to commercialize will be provided more and more in the future (Yang, Yu & Wu, 2020). The above content is often mastered by a small number of institutions, which is difficult to make the best use of. Video data transactions can break the dilemma of data islands (Taheri, Hariri & Rahmatollah Fattahi (2014)). Due to the reproducible characteristics of video, video is easy to be stolen in the process of transaction, and it is difficult for victims to protect their rights. At present, the development of data trading system is in the primary stage, and the system lacks unified standards and norms. Therefore, the video transaction lacks credible, safe and feasible implementation scheme. At present, API transactions and package transactions are the most popular video transaction methods on the Internet. API is the abbreviation of application programming interface. In short, API transactions allow two applications to exchange data in a predetermined format. API transactions can desensitize video transactions and prevent video from being stolen and reselled. However, the network transmission time is too long, this scheme can only be used for small file data transmission. If this scheme is used for large video file data transmission, the efficiency is very low. Although the transaction can be packaged to solve the problem of transaction file size, video data is easy to be easily lost, so it has not become the main solution in network transmission.

The existing video transaction mode has strong limitations. The video seller and the video buyer are isolated, and the relationship between them cannot be established directly, so the traditional trading mode cannot meet the demand of video trading. First of all, alliance blockchain technology uses cryptographic algorithms such as *Hash* to establish a trust relationship in the node cluster which lacks trust. This trust does not rely on any central party to make endorsement. In addition, the alliance chain is tamper proof and not traceable, which is suitable for video transaction application scenarios. Therefore, the alliance chain can be used to build video transaction index, trace the source of the traded video and identify the ownership of the video. Then, the technological innovation of trusted computing from the aspects of logical correctness verification, computing architecture and computing mode fundamentally improves the security of computer hardware and network environment, and reduces the possibility of hackers attacking the computer and its network that are trading.

This paper proposes a video transaction algorithm considering alliance chain and trusted computing. Based on alliance chain and trusted computing, and assisted by the algorithm of non encrypted symmetric system in cryptography, this paper hopes to solve the current transaction problems of video transactions: data can easily be stolen, the transaction environment is not secure and the transaction file size is limited. The specific objectives of the algorithm are as follows:

(1) The video seller uses the distributed storage method to store the original video file locally and obtain the video storage path. Then, based on the video digest calculation method, the digest file that can uniquely identify the video is calculated, and the hash value of the digest file is calculated through the *hash* function, which is used as the video index information and stored in the alliance blockchain.

(2) The buyer of video records the transaction process and the use of video through the alliance blockchain.

(3) Trusted computing technology ensures the security of the video trading environment, ensures that the video will not be leaked in the subsequent use process, and also ensures that the new data generated in the video use process will not be leaked.

### Related works

The blockchain proposed by Nakamoto (2009)’s in bitcoin white paper has attracted the attention of many researchers because of its characteristics of decentralization, tampering and transaction between untrusted nodes. Buterin (2014) proposes Ethereum, which introduces intelligent contract for the first time, which makes blockchain technology go out of the inherent application scenario of digital currency for the first time. Hyperledger fabric (Song, Kim & Kim, 2020) released by Linux foundation introduces member management service on the basis of blockchain, which provides solutions to blockchain enterprise applications.

### Application of blockchain technology in transaction

When blockchain is applied to transaction, experts and scholars have done a lot of research. Hu & Huang (2020) proposed a solution based on blockchain to solve the problem of low trust of agricultural products and lack of trust among members in the supply chain. Li, Li & Li (2020) constructed the “auction energy storage” contract system, designed the auction contract and energy storage contract, realized the unified management of user information, online transaction of energy storage idle capacity, “many to one” charge/discharge control of energy storage equipment and real-time settlement of transaction control cost. Chen, Zhao & Gong (2020) proposed a transaction model of electric vehicle charging based on blockchain. Under the market competition mechanism of “multi seller multi buyer”, it takes advantage of the characteristics of decentralization and high security of blockchain to restore the commodity attribute of electric power and open the right of users to negotiate pricing.

In the field of blockchain technology applied to video and other media transactions, Lin (2019) proposed an incentive mechanism and data storage scheme based on blockchain by combining P2P streaming media technology with blockchain technology to solve the problems of centralized data storage and centralization of incentive mechanism based on virtual currency in existing video sharing systems. Lu & Wang (2020) combined with Ethereum blockchain, interstellar file system, streaming media and front–end technology, proposed streaming media file sharing method and copyright protection method based on blockchain, and designed and developed a streaming media file sharing system to protect the rights and interests of creators. Jin, Wang & Dong (2020) takes the cloud blockchain platform + Software as a Service (SaaS) application platform + information service center as the overall architecture, and takes the regional radio and Television Alliance chain as the organizational basis to provide TOB and TOC digital media copyright deposit transaction management services. Shenyan & Xu (2019) adopts the alliance chain mode to elaborate the user role, data structure and contract process on the chain, forming the basic functional framework of copyright registration and transaction. Yin (2019) designed and implemented a digital content copyright registration and trading system based on blockchain, combined with blockchain, erasure correction code, web development and other technologies, starting from the needs of digital content copyright registration and trading.

### Application of trusted computing technology in transaction

There are many researches on the application of trusted computing technology in transaction. Zhang et al. (2020) proposed a data transaction scheme based on blockchain and trusted computing to solve the problems of data easy to be copied and data confidentiality in the current data transaction process. Liu & Xue (2020) proposed a trusted computing solution based on blockchain, which can effectively solve the problem of data sharing, break the data island, and solve the problem of users’ concern about data privacy disclosure to a certain extent. Sun, Yang & Gong (2020) proposed that trusted computing and blockchain, as an emerging technology in information security protection, can guarantee the safe and reliable operation environment and management mechanism in the distribution Internet of things.

Trusted computing is applied in the field of video media transaction. Zhou (2018) proposed a perception layer network access scheme based on behavior attributes for identity authentication of trusted computing. The scheme uses key distribution based on symmetric algorithm to complete bidirectional identity authentication, platform credibility authentication and behavior attribute authentication of cluster head node to perception node. Yue (2014) proposed a trusted digital rights model based on trusted computing, analyzed the basic information of trusted computing and trusted platform, and proposed a scheme of digital rights management combined with hardware protection. Jiang (2018) analyzed the characteristics and key technologies of trusted computing, and elaborated the application of trusted computing such as digital rights management, identity theft protection, preventing system harm and preventing game cheating.

The existing research team has done a very good job on TPM (trusted platform module), and the research results explain this problem very clearly. This paper will make further thinking based on the research results of this team (Mana & Munoz, 2006; Munoz & Mana, 2011b; Muoz, Maa & Serrano, 2009; Munoz, Mana & Serrano, 2009; Munoz et al., 2009; Maña & Muñoz, 2007; Mu & Fernandez, 2020; Muñoz & Lopez, 2018; Muñoz & Maña, 2011a; Muoz, Farao & Correia, 2020).

The existing video transaction scenarios have many disadvantages, such as high redundancy of the transaction system, lack of tracking of copyright information, and user privacy is easy to be eavesdropped. In this case, the importance of trusted computing is highlighted. This scenario can be used in trusted computing technology, such as authority control, authority delegation and direct anonymous authentication.

### *FISCO* alliance chain and improved trusted computing technology

#### *FISCO* alliance chain technology

**Figure 1:**
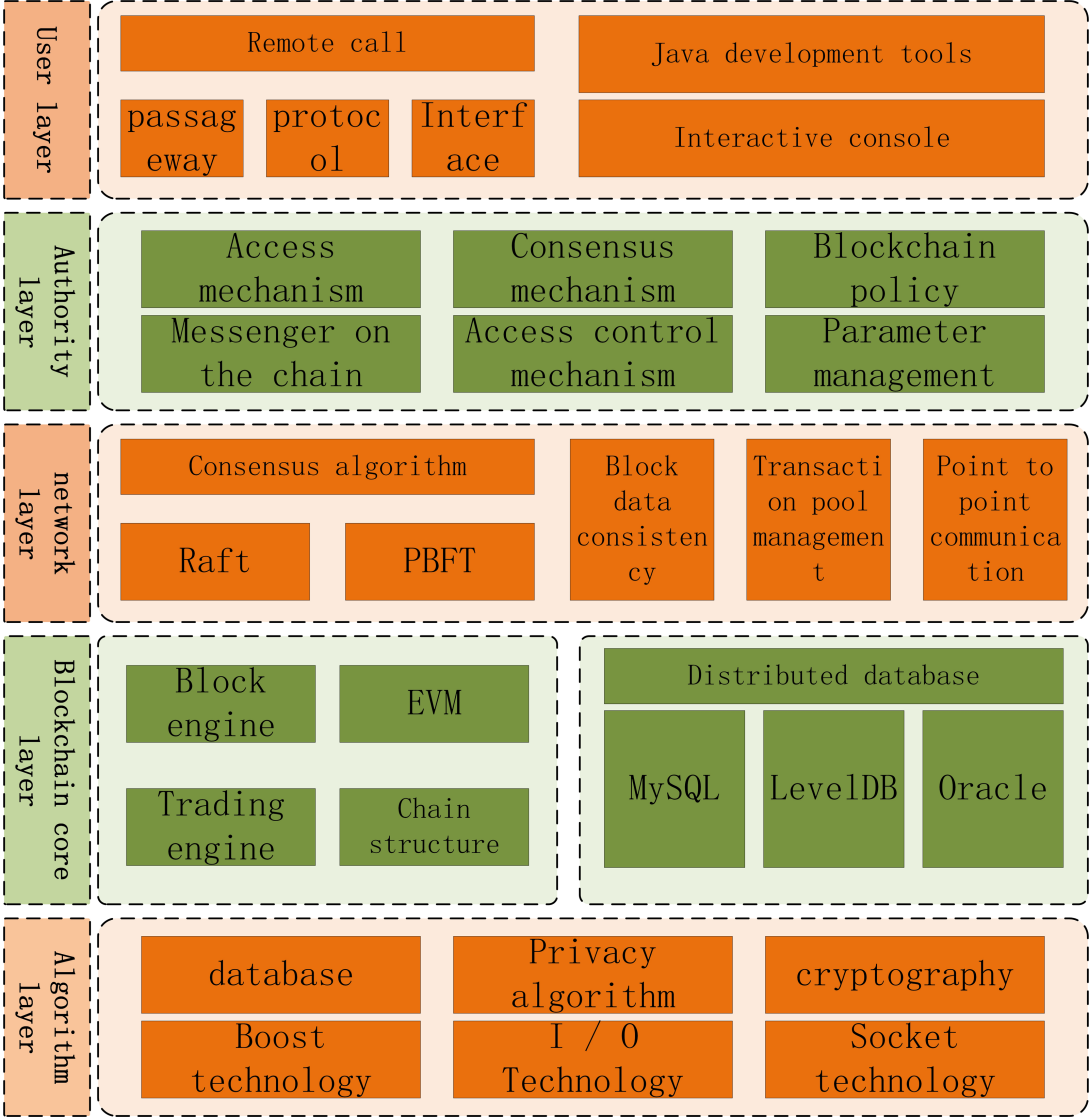
Divided from bottom to top into algorithm library layer.

*FISCO* bcos is a low-level blockchain platform independently developed and completely open-source by the open-source working group of *jinlianmeng*. The architecture of the platform is divided from bottom to top into algorithm library layer, blockchain core layer, network layer, permission layer and user layer, as shown in Fig. 1.

**(a) Key technology analysis**

**(1) Node access and permission control mechanism**

The public link in mechanism in the traditional blockchain technology allows any node to join the blockchain network at any time, and has the right to obtain the world state of the blockchain and all the data of the ledger from the global ledger. Any node in the alliance chain is not allowed to join the blockchain network without permission, and has a verification link.

Based on the above foundation, *FISCO* alliance chain technology does not adopt the traditional mechanism of “one account book for one chain”, but adopts its unique mechanism of “n account books for one chain”. This strategy realizes the data isolation of the same alliance chain in the dimension of group. All the members of the *FISCO* alliance chain will recognize a third-party management organization *manager* that can issue access mechanism authorization. When the *node* with *manager* authorization certificate logs in to the network of the *FISCO* alliance chain, it needs to initiate SSL two-way authentication operation. Through authentication, it can join the *FISCO* alliance chain. At this time, the traditional alliance chain will allow *node* to synchronize the alliance chain data and participate in consensus, so as to obtain the data on the chain. However, the *FISCO* alliance chain added an “access mechanism” before that, which requires *node* to request the system administrator’s authorization after successful login, and then become a node in the group. The permission of the group node is limited to “observation node” and “consensus node” Among them, the former can only participate in the synchronization of blocks in *FISCO* alliance chain, while the latter can participate in both synchronization and consensus initialization. The authorization of this permission depends on the system administrator.

**(2) Parallel computing**

Traditional blockchain transaction performance is low. The parallel transaction processing model of FISCO alliance chain can make the transactions in the block execute in parallel, which improves the performance of transaction execution. FISCO provides users with the interface to write parallel contracts and the environment to execute parallel transactions. Developers define each interface in Le contract by defining mutually exclusive parameters.

**(3) Distributed storage technology**

**Figure 2:**
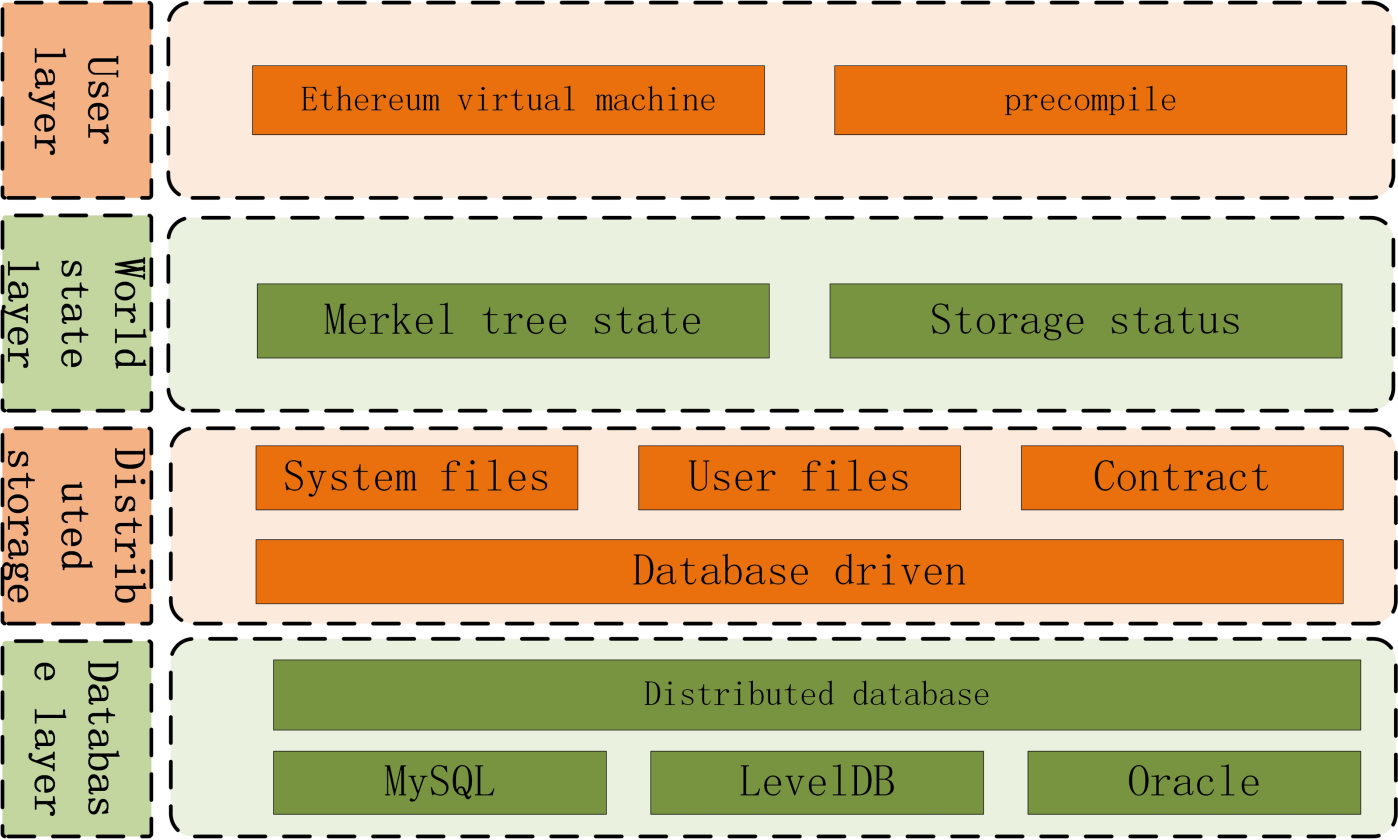
Storage model architecture.

Traditional Merkel tree storage has performance bottleneck. *FISCO* redefines the underlying storage model of blockchain and supports *key-value* database. The storage model architecture is shown in Fig. 2.

**(4) Consensus algorithm**

For different application scenarios, *FISCO* supports two different consensus algorithms: practical Byzantine fault tolerance algorithm and raft consensus algorithm. The former uses cryptography algorithm to ensure the anti-counterfeiting, anti tampering and non repudiation in the process of message delivery. The fault tolerance is *3f+1*, in which *f* represents the number of malicious nodes. As mentioned above, the parallel computing capability of *FISCO* will make all nodes run in parallel as far as possible, and optimize the critical path to reduce the time loss in the consensus process, so as to improve the efficiency of the practical Byzantine fault-tolerant algorithm.

In the latter, each node competes fairly in the time window to obtain accounting rights, and the fault tolerance is *2f+1*, where *f* represents the number of malicious nodes. *FISCO* alliance chain optimizes the network delay and jitter, and raft consensus algorithm can get better robustness in extreme network environment. Combined with smart contract, it realizes the dynamic in and out of nodes.

**(a) Key feature analysis**

**(1) Security features**

The communication between all nodes in *FISCO* alliance chain is based on *SSL* protocol. Moreover, the access mechanism of nodes, the approval mechanism of administrators and the distributed permission control mechanism also ensure the security of *FISCO* alliance chain. Among them, admission mechanism and recognition mechanism not only break the connection between *FISCO* alliance chain and malicious nodes. Moreover, the blacklist is set, which fundamentally eliminates the possibility of the node attacking again. Access control mechanism strictly controls the access of sensitive data. In addition, the *FISCO* alliance chain also introduces homomorphic encryption and zero knowledge proof mechanism to further improve the security.

**(2) Available features**

*FISCO* alliance chain simplifies the process of building alliance chain and reduces the standard of deployment mode. In addition, fsico alliance chain supports multiple programming languages and SDK interfaces, which reduces the development threshold and can better adapt to complex and changeable application scenarios, and improves the availability of *FISCO* alliance chain.

**(3) Performance features**

The transaction concurrent execution model of *FISCO* alliance chain ensures that the system automatically constructs *DAG* of transaction dependency according to mutually exclusive variables and transaction order in the transaction, and then uses *DAG* to execute transactions concurrently as much as possible, so as to improve the transaction processing speed in the block. *FISCO* alliance chain supports *C + +* to write smart contracts, so it can achieve about 20,000 *TPS* per chain. In addition, the support of *FISCO* alliance chain for multi group architecture is reflected in the process of ledger data sharing and consensus. It can improve the system throughput per unit time and the performance of the alliance chain.

### Improving trusted computing technology

**Figure 3:**
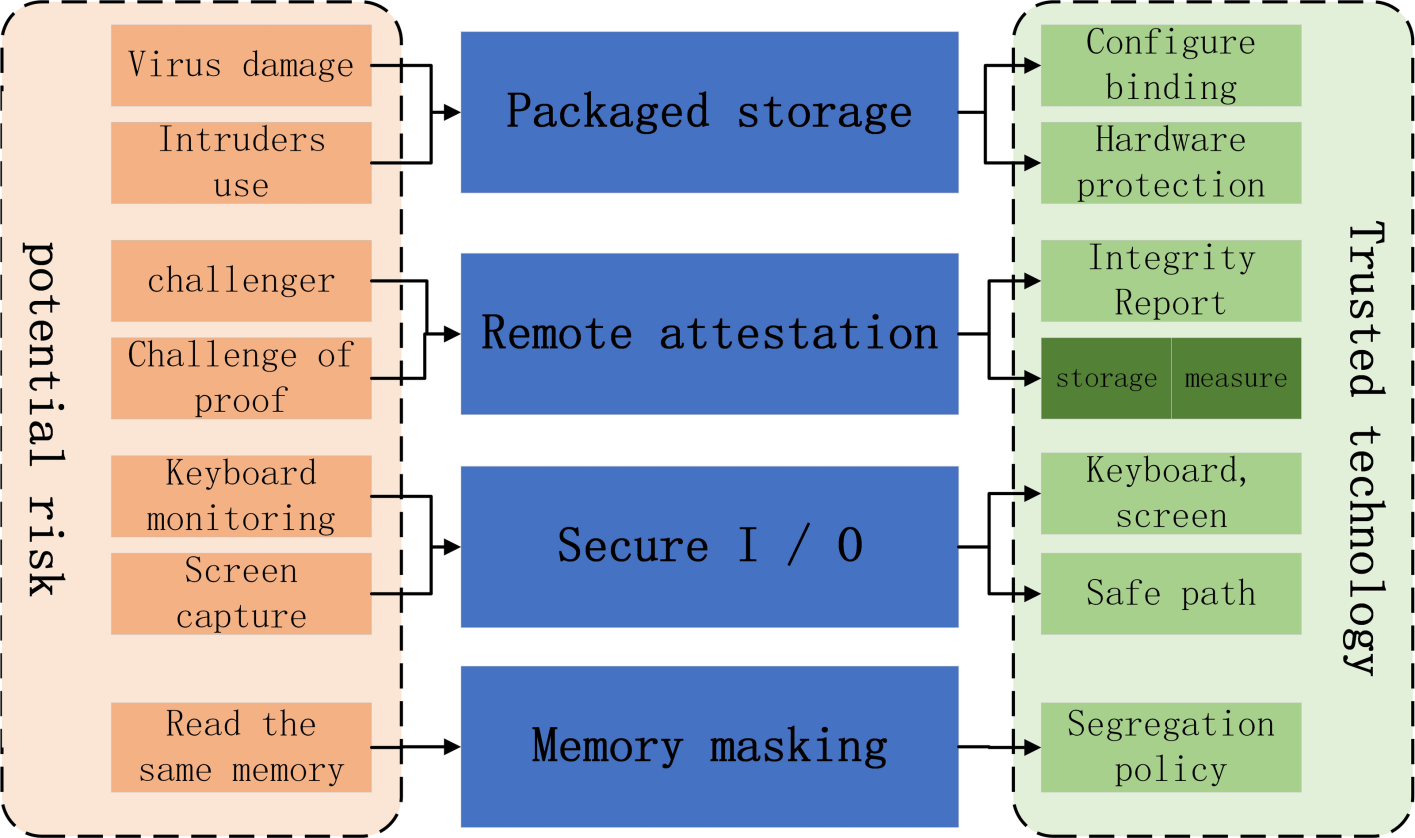
The entity’s characteristics.

In the early research, trusted computing technology focused on the security of the operating system. With the rise of Internet of things, big data and cloud computing, the research scope of trusted technology is expanding. If the behavior of an entity is always carried out in the expected way and towards the expected goal, then the entity is considered to be credible, and its characteristics are shown in Fig. 3.

(1) Trusted computing will provide reliable protection on the hardware level for encrypted information. The method is to bind the encrypted information with the software configuration. If the configuration value given by the software accessing the encrypted information does not conform to the standard, the system will not release the encrypted information to resist hackers and viruses. This feature is called “encapsulated storage”;

(2) The method is that the Challenger sends the challenge information to the *prover*, and the *prover* uses the AIK (attachment identity key) of the TPM, AIK) signature is fed back to the challenger, and the Challenger verifies the integrity or running state of the platform by comparing the expected value with the signature value. The feature that “one running program can send the verification challenge information to another running program to verify its integrity or running state” is called “remote proof”;

(3) Trusted computing will provide protection against hackers’ attacks by monitoring keyboard input information and capturing screen display information. The method is to provide a secure channel from “keyboard to program” and “program to screen”, which is called “secure I/O”;

(4) Trusted computing will provide a means of shielding against attacks launched by different programs reading the same memory. The method is to make different programs read memory information in isolation. This feature is called “memory shielding”.

This paper improves the trusted computing technology by fusing the traditional zero knowledge proof and improving the identity authentication mechanism, and analyzes the improved trusted computing technology from multiple dimensions.

**Figure 4:**
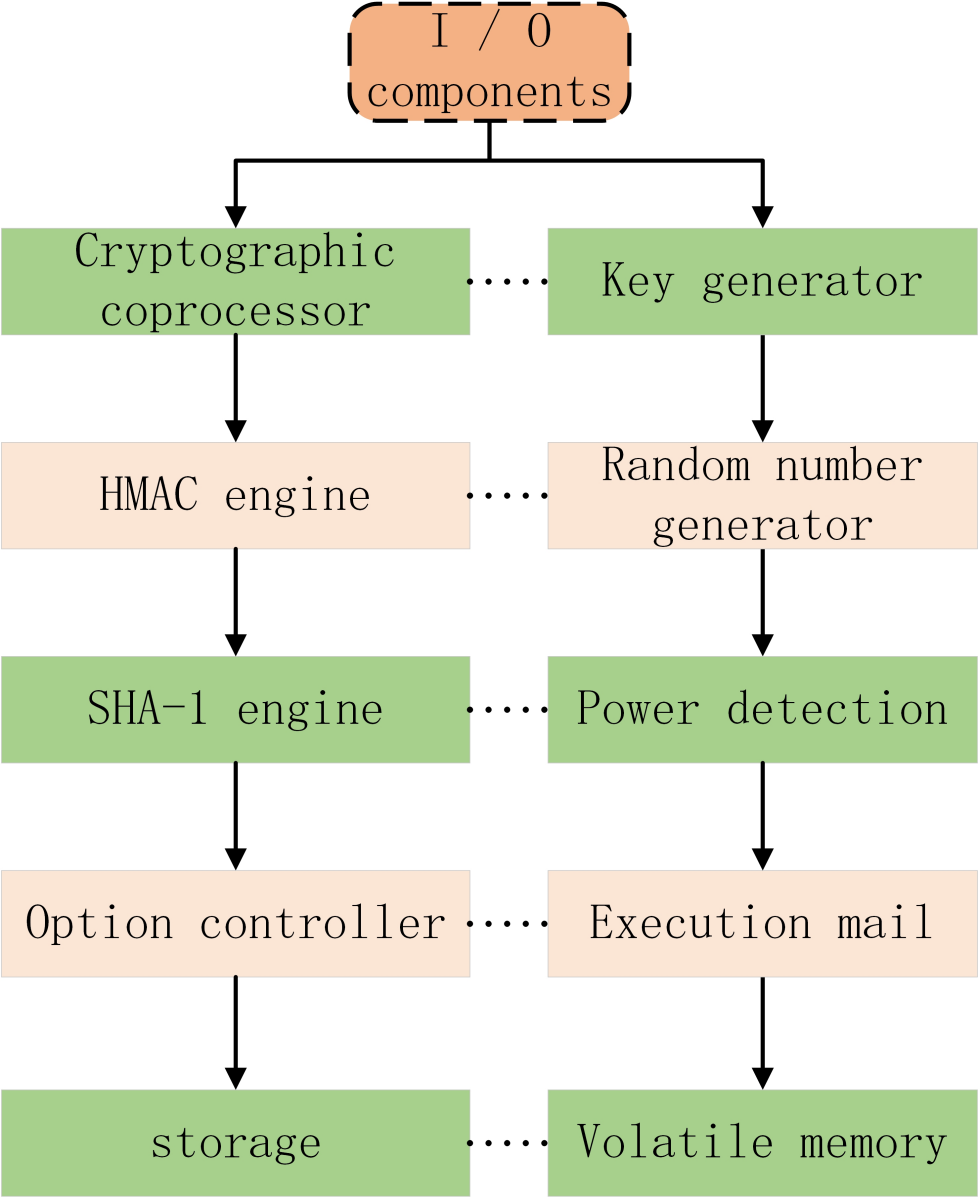
Specific composition of TPM.

The core of trusted computing is Trusted Platform Module TPM. Trusted platform module is a kind of hardware core module integrated in trusted computing platform, which is used to establish and guarantee trust source. It provides integrity measurement, secure storage, trusted report, platform authentication and control, password service and other functions for trusted computing. Usually, TPM is embedded on the main board of computer, which provides services for DRM system by using hardware characteristics. The specific composition of TPM is shown in Fig. 4.

I/O component: is mainly responsible for managing the information flow on the communication bus, executing the communication protocol between the internal bus and the external bus, and sending messages to the appropriate components.

Key generator: responsible for generating the key pair required by TPM. For RSA algorithm, it needs to complete the test of large prime number, and the key generation process will use the data randomly generated by random number generator.

HMAC engine: it provides information for TPM by confirming whether the message data is correct or not. It can find that the data or command is wrong or tampered.

Random number generator: it is responsible for generating random numbers needed by various operations. It transforms an unpredictable input into a 32 byte random number through an internal state machine and one-way hash function.

Power detection: help TPM take appropriate limiting measures when power state changes.

Option controller: provides the mechanism to turn on and off the TPM function. By changing some permanent variable flag bits, you can set the TPM function options.

Executive part: it is responsible for executing the commands sent to TPM through I/O. before executing the commands, it should be sure that the command execution environment is isolated and safe.

Nonvolatile memory: used to store some permanent data.

**(a) Zero knowledge proof**

Zero knowledge proof means that *prover* P proves to *verifier* Q that he knows information I, but the proof process cannot disclose any information of I. its essence is that N participants perform a task according to certain rules. Its characteristics are as follows

(1) Certainty: if P doesn’t know information I, he can’t convince Q that he will prove it;

(2) Integrity: if P knows information I, then P can make Q believe that he will prove it;

(3) Zero knowledge: Q cannot acquire any additional knowledge about information I.

The cave model, the most typical zero knowledge proof case in trusted computing technology, is shown in Fig. 4. There is a secret door between point C and point D, which can only be opened by knowing the secret order. If the *prover* P knows the secret order and wishes to prove it to the *verifier* V, but does not wish to disclose it, then:

(1) First, the verifier V stands at point A and the prover P stands at point B;

(2) Prover P randomly chooses to go to point C or D, and verifier V can’t see the direction of prover P at point A;

(3) The verifier V goes to point B and asks the verifier P to come out from the left/right channel;

(4) The prover P comes out from the designated direction according to the requirements of the verifier V. if necessary, he needs to use the secret order to open the secret door.

If the prover P knows the secret order, he will be able to come out correctly from the direction required by the verifier V.

In the application of blockchain, the data interaction verification of different participants can be realized by zero knowledge proof, which can avoid the mutual leakage of sensitive information; in the application of multi-party computing, participants can ask each other to provide zero knowledge proof results of the calculation process for verification after the privacy calculation protected by homomorphic encryption and other methods is completed. This method prevents the false calculation, and at the same time does not disclose the sensitive information in the calculation.

**(b) Improve identity authentication mechanism**

The direct anonymous authentication protocol proposed by the trusted computing organization solves the problem of low efficiency of the private CA (certificate authority) protocol, but it also reduces the privacy. Therefore, based on the above zero knowledge proof, the double random number signature algorithm based on Elgamal encryption strategy will be innovatively used in the signature phase to authenticate the trusted anonymous identity.

Suppose that *pri* is a large prime in the finite field *GF(pri)*, and set *Z*_*pri*_ and ${Z}_{pri}^{\ast }$, as shown in Formula (1) and Formula (2).

(1) \begin{eqnarray*}& {Z}_{pri}=\{0,1,2,\ldots ,pri\}\end{eqnarray*}

(2) \begin{eqnarray*}& {Z}_{pri}^{\ast }=\{0,1,2,\ldots ,pri-1\}\end{eqnarray*}

The random number *ran* is selected from the subset ${Z}_{pri}^{\ast }$, and the maximum common divisor of the random number and the large prime *pri* is 1, as shown in Formula (3).

(3) \begin{eqnarray*}gcd(ran,pri)=1\end{eqnarray*}

In the definition of public and private key, the private key is *pri_key*, the public key is *pub_key*, as shown in Formula (4) and Formula (5).

(4) \begin{eqnarray*}& pri\text{_}key\in {Z}_{pri}^{\ast }\end{eqnarray*}

(5) \begin{eqnarray*}& pub\text{_}key=ra{n}^{pri\text{_}key}\mathrm{mod} pri\end{eqnarray*}

The *user* seeks the verification of the signed message *mes* from the verifier *verd*, and the specific steps are as follows:

(1) *User* selects the random number *ran*′ from the subset ${Z}_{pri}^{\ast }$, so that the random number and the large prime *pri-1* satisfy the greatest common divisor of 1, as shown in Formula (6).

(6) \begin{eqnarray*}gcd(ra{n}^{{^{\prime}}},pri-1)=1\end{eqnarray*}

(2) The middle number *mid* is calculated as shown in Formula (7).

(7) \begin{eqnarray*}mid=ra{n}^{ra{n}^{{^{\prime}}}}\mathrm{mod} pri\end{eqnarray*}

(3) Calculate the temporary variable *tem* in the congruence equation, as shown in Formula (8) and Formula (9).

(8) \begin{eqnarray*}& mes\equiv (pri\text{_}key\cdot mid+ra{n}^{{^{\prime}}}\cdot tem)mod(pri-1)\end{eqnarray*}

(9) \begin{eqnarray*}& tem\equiv (mes-pri\text{_}key\cdot mid)\cdot ra{n}^{{^{\prime}}-1}\mathrm{mod} (pri-1)\end{eqnarray*}

The *user’s* signature on *mes* is *(mid, tem)*, and *user* sends the request verification message *req* to *verd*, as shown in Formula (10).

(10) \begin{eqnarray*}req=(mes,mid,tem)\end{eqnarray*}

After *verd* receives *req*, it first verifies whether *mid* is in the closed interval [*1,pri-1* ], if not, it directly rejects the signature; otherwise, it continues to calculate to verify whether the equation *ran*^*mes*^ is true, as shown in Formula (11).

(11) \begin{eqnarray*}ra{n}^{mes}\equiv pub\text{_}ke{y}^{mid}\cdot mi{d}^{tem}\mathrm{mod} pri\end{eqnarray*}

If it is not true, the signature will be rejected directly, otherwise the signature will be verified to be true and valid. But the above scheme only uses a random number *ran*′, if the random number is stolen by the attacker, the above calculation cannot guarantee the security. On this basis, this paper proposes a double random number signature authentication mechanism to improve the identity authentication mechanism in trusted computing:

(1) User again selects the random number *ran*^∗^ from the subset ${Z}_{pri}^{\ast }$, which satisfies the greatest common divisor of 1 with the large prime *pri-1*, as shown in Formula (12) and Formula (13).

(12) \begin{eqnarray*}& ra{n}^{{^{\prime}}{^{\prime}}}\not = ra{n}^{{^{\prime}}}\end{eqnarray*}

(13) \begin{eqnarray*}& gcd(ra{n}^{{^{\prime}}{^{\prime}}},pri-1)=1\end{eqnarray*}

(2) Calculate the intermediate number *mid*′, as shown in Formula (14).

(14) \begin{eqnarray*}mi{d}^{{^{\prime}}}=ra{n}^{ra{n}^{{^{\prime}}{^{\prime}}}}\mathrm{mod} pri\end{eqnarray*}

(3) Calculate the temporary variable *tem*′ in the congruence equation, as shown in Formula (15).

(15) \begin{eqnarray*}mes\equiv (pri\text{_}key\cdot mid+ra{n}^{{^{\prime}}}\cdot mi{d}^{{^{\prime}}}+ra{n}^{{^{\prime}}{^{\prime}}}\cdot tem)\mathrm{mod} (pri-1)\end{eqnarray*}

(4) The verification equation *ran*^*mes*^ is calculated as shown in Formula (16).

(16) \begin{eqnarray*}ra{n}^{mes}\equiv pub\text{_}ke{y}^{mid}\cdot mi{d}^{mid}\cdot mi{d}^{{^{\prime}}tem}modpri\end{eqnarray*}

The *user’s* signature on *mes* is (*mid*, *mid*′, *tem*), and *user* sends the request verification message *req* to *verd*, as shown in Formula (17).

(17) \begin{eqnarray*}req=(mes,mid,mi{d}^{{^{\prime}}},tem)\end{eqnarray*}

**Figure 5:**
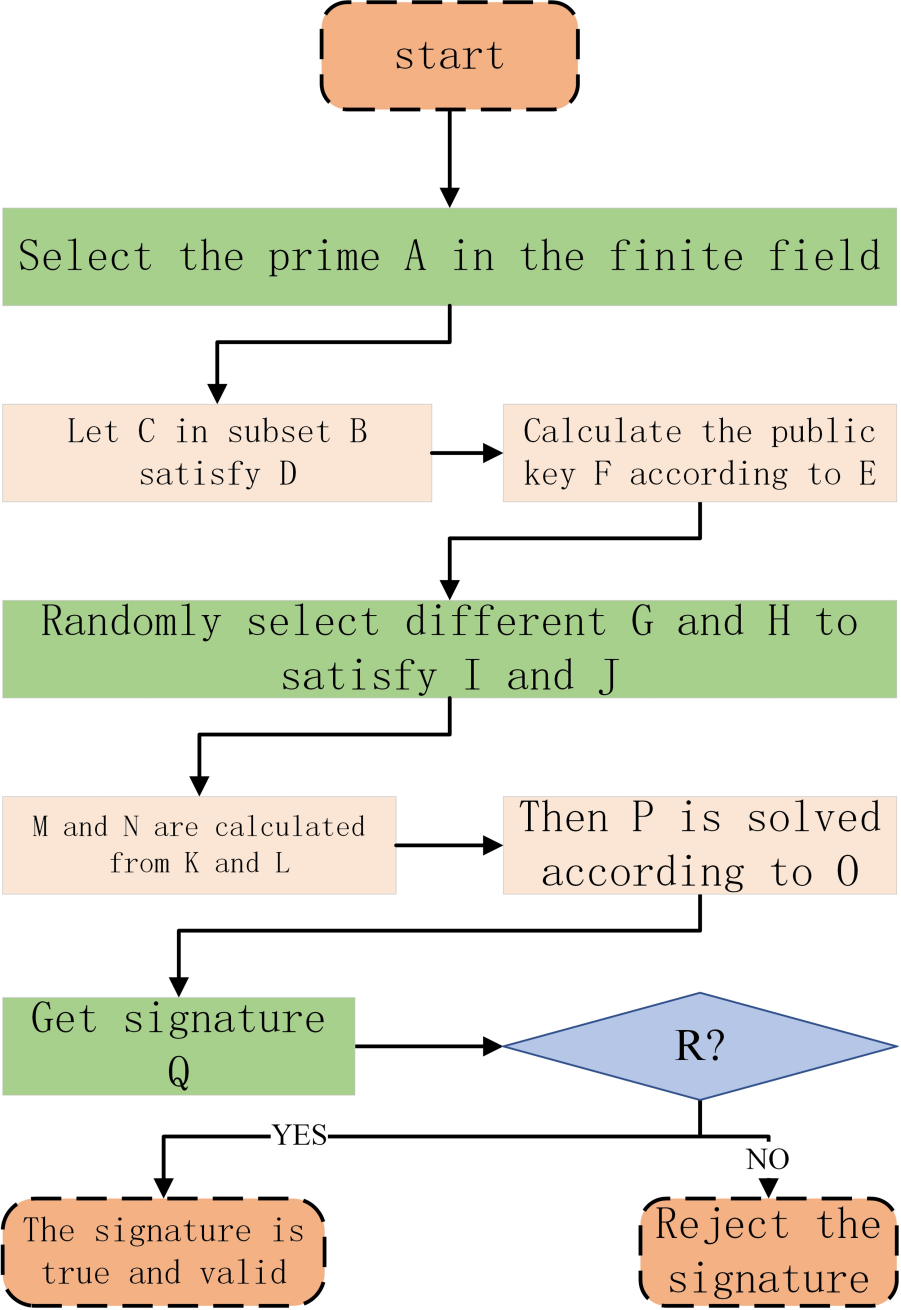
Above Trusted anonymous authentication process.

After *verd* receives the *req*, it first verifies whether *mid* and *mid*′ are in the closed interval [*1,pri-1* ], if not, it directly rejects the signature; otherwise, it continues to calculate whether the verification equation *ran*^*mes*^ is true, if not, it directly rejects the signature, otherwise, it verifies that the signature is true and valid. The above trusted anonymous authentication process is shown in Fig. 5, and the letter Formula correspondence in the flow chart is shown in Table 1.

**Table 1:** Flowchart letter-Formula correspondence table.

A	*pri*	B	${Z}_{pri}^{\ast }$
C	*ran*	D	gcd(*ran*, *pri*) = 1
E	*pub*_*key* = *ran*^*pri*_*key*^mod*pri*	F	*pub_key*
G	*ran*′	H	*ran*^^′′^^
I	gcd(*ran*′, *pri* − 1) = 1	J	gcd(*ran*^^′′^^, *pri* − 1) = 1
K	*mid* = *ran*^*ran*′^mod*pri*	L	*mid*′ = *ran*^*ran*^^′′^^^mod*pri*
M	*mid*	N	*mid*′
O	*mes* ≡ (*pri*_*key*⋅*mid* + *ran*′⋅*tem*)mod(*pri* − 1)		
P	*tem* ≡ (*mes* − *pri*_*key*⋅*mid*)⋅*ran*′^−1^mod(*pri* − 1)		
Q	*req* = (*mes*, *mid*, *mid*′, *tem*)		
R	*ran*^*mes*^		

**(c) Technical analysis**

Trusted computing is frequently used in untrusted transactions. This paper will analyze the improved trusted computing.

**(1) Security—attackers steal signatures**

Assuming that the attacker successfully steals *mid* and *mid*′ in the signature, the paper proposes a more secure double random number signature authentication mechanism, which makes the verification equation change from Formula (18) to Formula (19). Because of the extra inverse operation, the complexity of the algorithm increases exponentially. Even if the attacker steals the signature, he can only attack the stolen information, but cannot attack other messages.

(18) \begin{eqnarray*}& mi{d}^{tem}\equiv pub\text{_}ke{y}^{-mid}\cdot ra{n}^{mes}\mathrm{mod} pri\end{eqnarray*}

(19) \begin{eqnarray*}& mi{d}^{{^{\prime}}tem}\equiv pub\text{_}ke{y}^{-mid}\cdot ra{n}^{mes}\cdot mi{d}^{imid}\mathrm{mod} pri\end{eqnarray*}

**(2) Security—attacker associated random number**

If the random number *ran*′ selected by two successive signatures is the same, then Formula (7) shows that the intermediate number *mid* is the same, and then Formula (15) can calculate the random number *ran*^′′^, so the security cannot be guaranteed, so the random number generated each time must be random enough; in addition, if there is a simple addition and subtraction operation relationship between the random numbers selected by two signatures, the impact is small, because Formula (7) shows that The middle number *mid* is different, and then the *N*(*N* > 1) equations are calculated according to Formula (15), and only a few random numbers can be obtained, which ensures the security of trusted computing from the side.

**(3) Security—attacker steals private key**

Because of the complexity of the congruence equation in Formula (15), if we only know the parameter *primary_key*, we cannot calculate the random number *ran*′. This kind of problem of solving discrete logarithm in the finite field cannot guarantee the unique calculation of the random number *ran*′ and *ran*^′′^. Even if we calculate the solution set, it is almost impossible to complete the verification by substituting it into the congruence equation, so the security can be guaranteed.

**(4) Anonymity**

In the process of authentication, the algorithm uses zero knowledge proof, and never publishes the private identity information. In the signature stage, it also uses the double random number signature authentication mechanism with the same anonymity. In the signature information (*mid*, *mid*′, *tem*), *mid* and *mid*′ are calculated by random numbers *ran*′ and *ran*^′′^, so the signature is unique and unpredictable, which ensures the anonymity of trusted computing.

**(5) Computational complexity**

In order to ensure the security of random number calculation after being stolen by attackers, this paper proposes a double random number signature authentication mechanism based on single random number calculation, and adds *mid*′ shown in Formula (14) to the signature information. m represents a congruence equation, *m* ≡ (*xr* + *kλ* + *ts*)*mod*(*p* − 1). In this paper, two large prime numbers, P and Q, are randomly selected in a credible way, and then the value of *n* is calculated by *n* = PQ. Because this paper uses double random number signature, the traditional algorithm only uses one random number. Therefore, the operation of random number in double random number signature will be calculated once more.

In addition, compared with the traditional algorithm, the calculation of *g*^*m*^ ≡ *y*^*r*^*r*^*λ*^*λ*^*s*^*modp* becomes more complex and increases the amount of calculation. *λ* = *g*^*t*^*modp* is added to the signature, but the time will not change much. So the time complexity can be calculated. The above operation increases the computational complexity, but the time complexity *T*(*n*) does not change, as shown in Formula (20).

(20) \begin{eqnarray*}T(n)=ln mes {ln}^{2}n+{ln}^{2}m+{n}^{2}\end{eqnarray*}

To sum up, the performance of the improved trusted computing technology proposed in this paper is better than that of the traditional trusted computing technology in terms of security and anonymity, and it is no more complex than the traditional trusted computing technology in terms of computational complexity, especially in terms of time complexity. Therefore, the improved trusted computing technology proposed in this paper is feasible. This paper have proposed that the double random number signature method proposed in this paper will perform one more operation, but the change of time is small, which can still guarantee the original time complexity.

### Video transaction algorithm considering *FISCO* alliance chain and improved trusted computing

The proposed algorithm is mainly composed of trusted computing preparation, transaction model permission control, key storage migration, video abstract information extraction, and video transaction based on FISCO alliance chain. Through trusted identity authentication and DRM use control of FISCO alliance chain node, video summary information can be traded on the chain.

### Preparation of trusted computing environment for transactions

The node of video transaction based on *FISCO* alliance chain cannot resist the Byzantine attack without considering the non Byzantine attack initiated by the attacker, so the trusted authentication mechanism is essential in a secure and perfect transaction system. In addition, the transfer of video digest information is often accompanied by the transaction between the members of *FISCO* alliance chain, so it is necessary to prepare a trusted computing environment for the transaction. In this paper, the preparation method of trusted computing environment for transaction is divided into two stages, which are transaction model permission control method and key storage migration method.

**(a) Access control method of transaction model**

In the traditional research field of copyright protection and privacy protection, the subject with its own unique behavior attribute can have partial authority to control the object, and the subject can take corresponding deployment decisions according to the behavior attribute. In addition, the transaction model permission control method also includes inherent permission control and user permission control.

**(1) Inherent authority control method**

This control method stipulates that the use of video abstract information by all the member nodes based on *FISCO* alliance chain should be within the scope of the license file *all_license* of the alliance chain, and the license of the alliance chain, as the core license file of the whole alliance chain, can Formulate the specific permission of video abstract information through the contract. In this paper, the license is defined by the right description language XrML It is unique and universal. The administrator can describe the information of the buyer or seller node freely and normatively, including the user permission information.

**(2) Method of user authority control**

This control method first authenticates all the member nodes entering the trading system in the form of password, password, fingerprint, etc. only the user who has passed the authentication has the right to access and operate the trading system, and the system can track the user’s usage log.

**(b) Key storage migration method**

If the member node in *FISCO* alliance chain needs to replace the hardware device bound with the key due to irresistible reasons or non Byzantine errors, the new hardware device must have a strong robustness and fault-tolerant key storage migration method to ensure that it can continue to use the completed video.

**(1) Key storage method**

**Figure 6:**
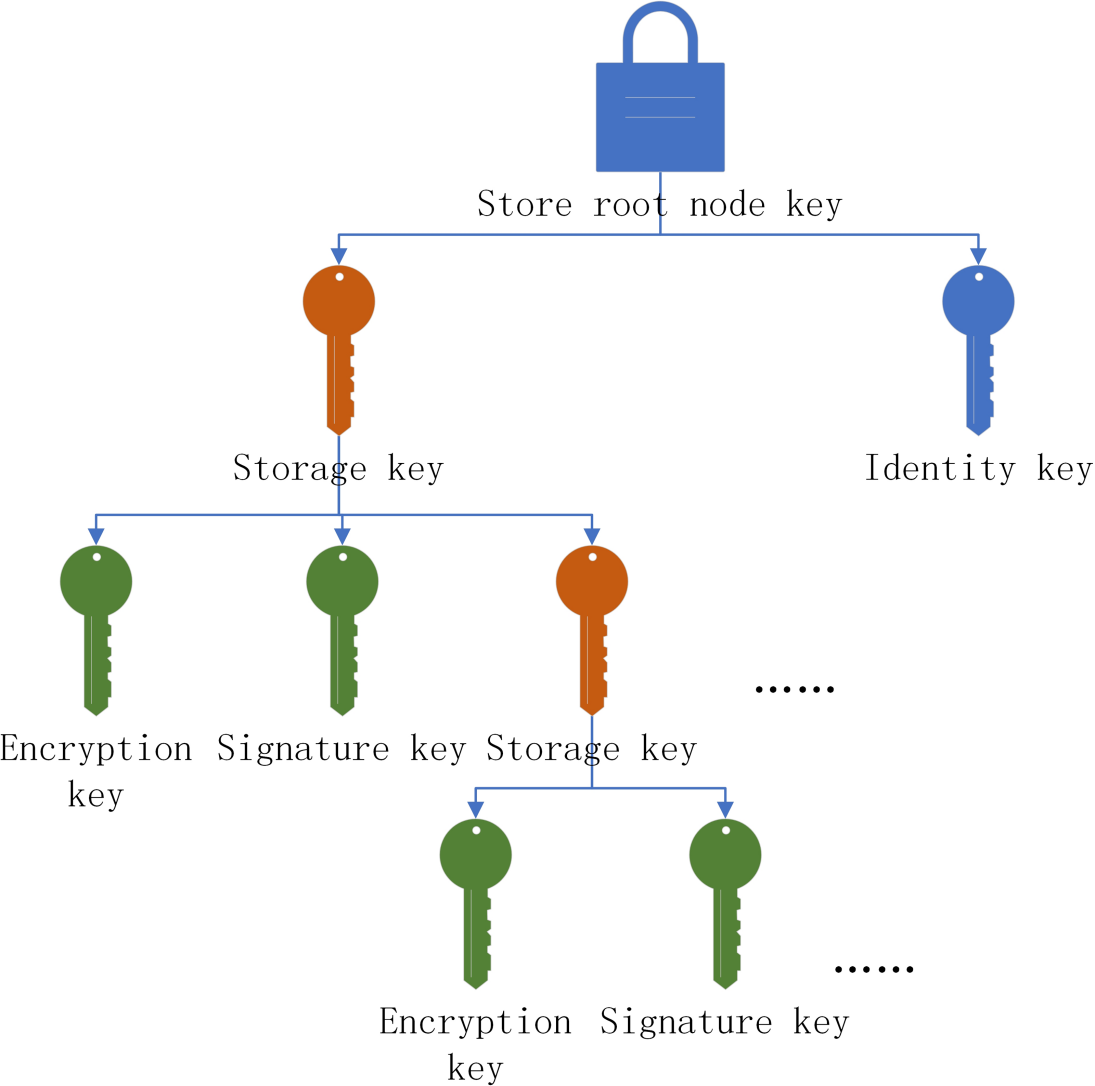
The loss of all the keys.

The trusted platform includes binding key, signature key, legacy key, migration key, storage key and identity key. Due to the limited storage capacity of the trusted platform, the platform is not enough to store all the keys at the same time. Therefore, this paper considers the combination of external storage and platform module storage. Therefore, the concept of parent key is introduced. The parent key storage system is similar to *Merkleroot*. The parent key starts from the root node. The storage root node is the core of the system, and the exposure of the root node means the loss of all the keys, as shown in Fig. 6.

**(2) Key migration method**

The authorization value of the key is determined by the user, and the certificate of the trusted platform module is randomly generated by the *TPM_TakeOwnership()* function and stored in the trusted platform module. Therefore, when the key is migrated, the certificate of the trusted platform module cannot be provided and the migration operation cannot be completed.

**Figure 7:**
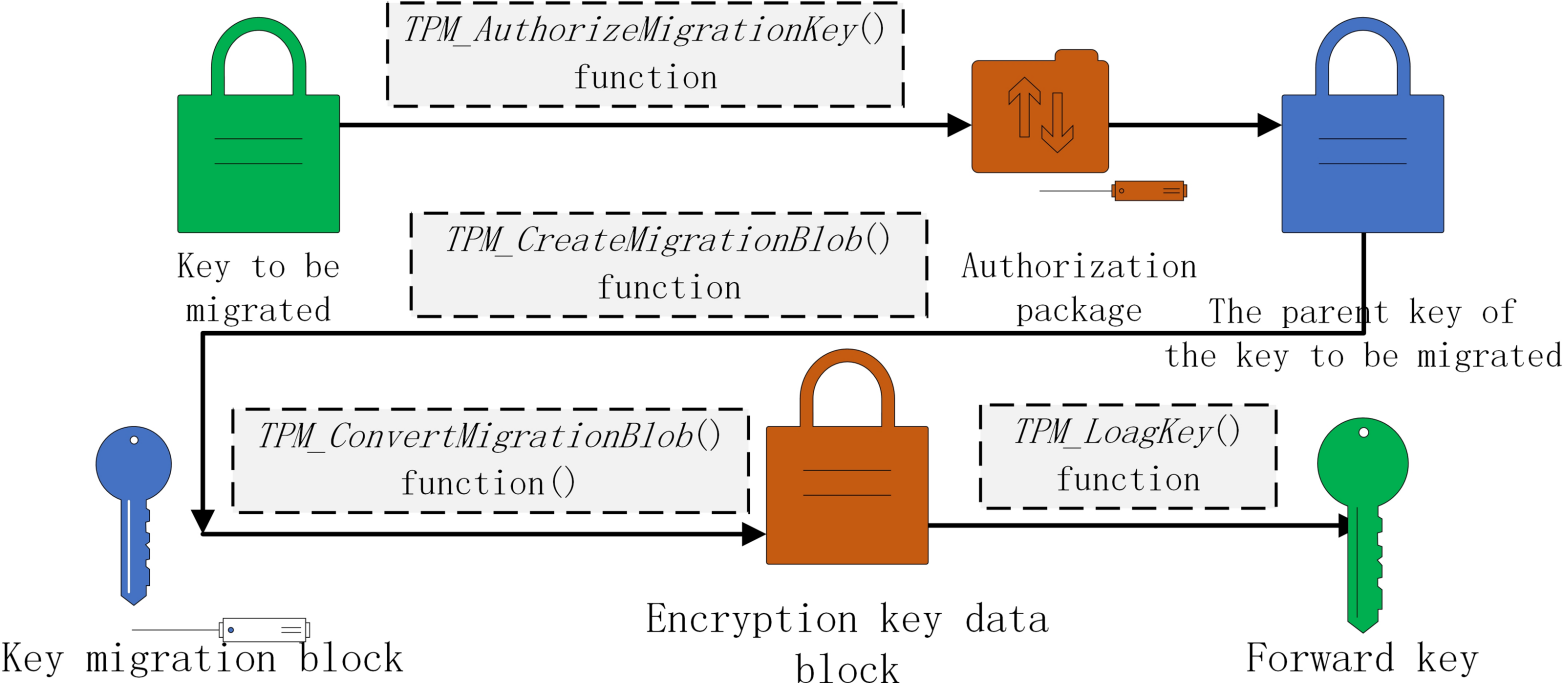
The transaction algorithm is divided (from top to bottom) into the following layers: user, service, core, and foundation.

The trusted platform module uses *TPM_AuthorizeMigrationKey()* function to authorize the migration and protect the key, generates the authorization package and obtains the authorization information, and then creates the key migration block through *TPM_CreateMigrationBlob()* function. After receiving the key migration block, the new trusted platform module converts it into a new platform key block through *TPM_ConvertMigrationBlob()* function, and finally loads the new key into the trusted platform module using *TPM_LoagKey()* function to complete the key migration. The process is shown in Fig. 7.

### Video Abstraction Algorithm

To prevent *DoS* attacks, bitcoin sets the block size to *1MB/block*. This regulation limits the possibility of large files trading on the blockchain. Therefore, this paper uses *Merkle* tree to collect transactions and package them. In the algorithm proposed in this paper, each transaction will go through different processes from the generation of the client to the sending of the signature to the nodes in the alliance chain. In order to improve the efficiency of the transaction, this paper uses the video abstract information which can uniquely identify the video as the transaction object.

**(a) Analysis of video abstract information**

**Figure 8:**
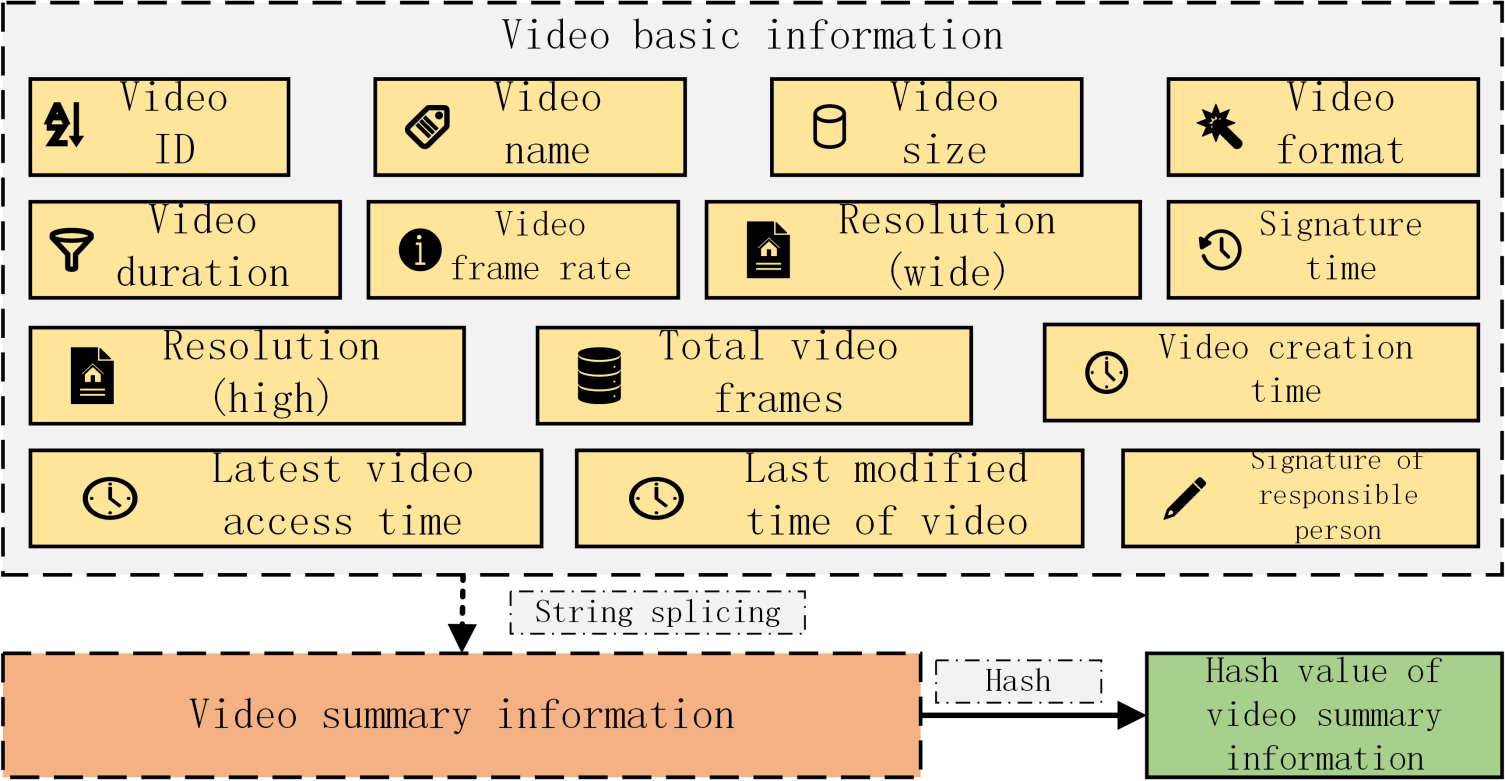
*Hash* value of video abstract information for transaction.

This paper proposes a video transaction algorithm considering *FISCO* alliance chain and improved trusted computing, which allows video transaction between member nodes joining the alliance chain. The object of transaction is the video summary information of the traded video, including the video ID automatically generated by the local database of the video seller, the video name, video size, video format and video content provided by the video seller Frequency duration, video frame rate, resolution (wide), resolution (high), total video frames, video creation time, video latest access time, video latest modification time, responsible person signature and signature time. All the above information is expressed in string form, and finally connected into a long string that can uniquely represent the video. In order to facilitate the processing of video abstract information, this paper uses *Hash* based on sha256 to calculate the *Hash* value of video abstract information for transaction, as shown in Fig. 8.

**(b) Video abstract information extraction**

**Figure 9:**
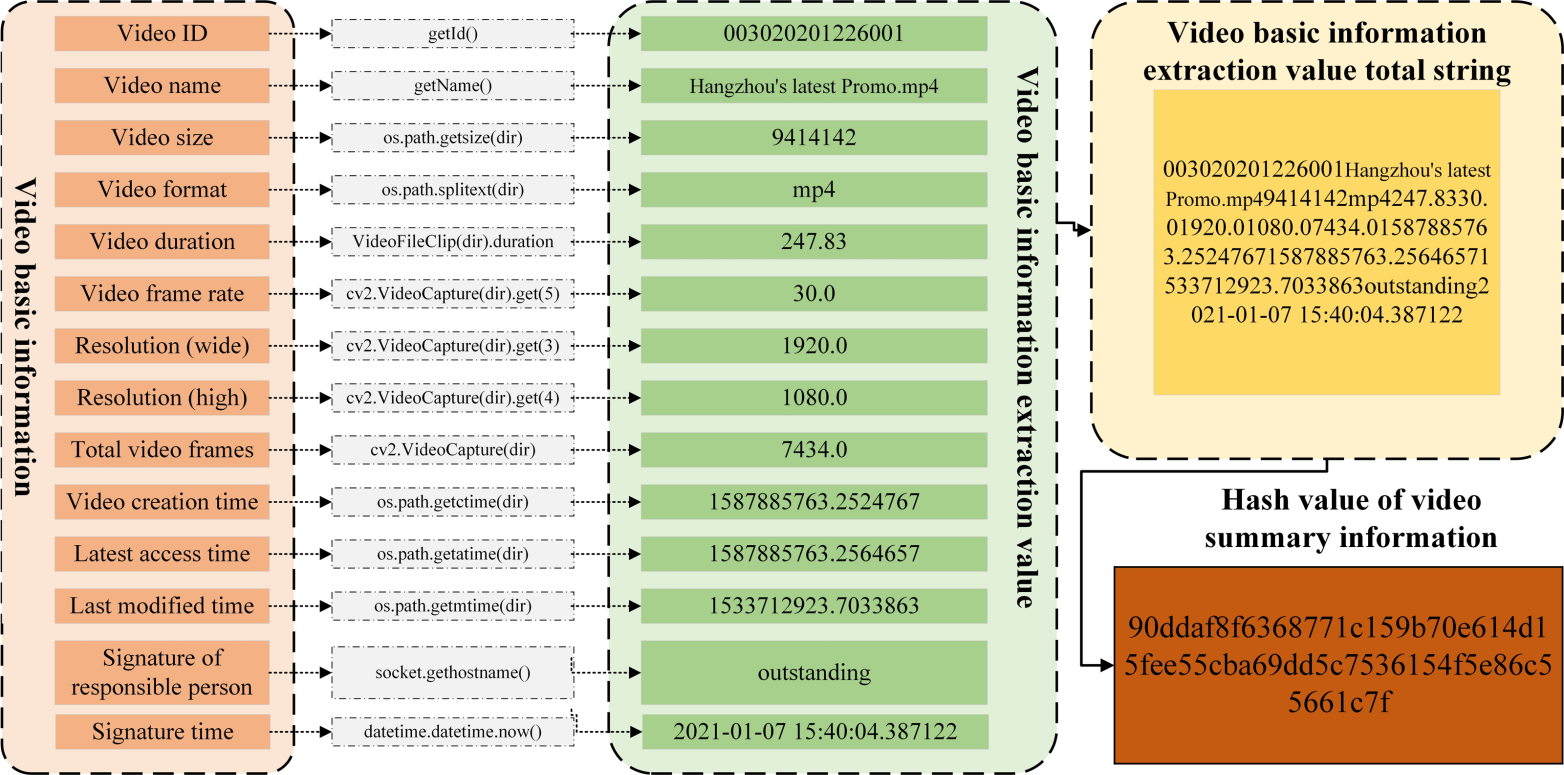
The schematic diagram of video abstract information.

In order to improve the efficiency of video transaction based on *FISCO* alliance chain, member nodes trade the *Hash* value of video abstract information, so this paper uses OpenCV, a cross platform computer vision and machine learning software library licensed by BSD, to extract video abstract information. As shown in Fig. 9, which is the schematic diagram of video abstract information extraction for video “*Hangzhou’s latest Promo”*.

### Video transaction algorithm based on *FISCO* alliance chain

**Figure 10:**
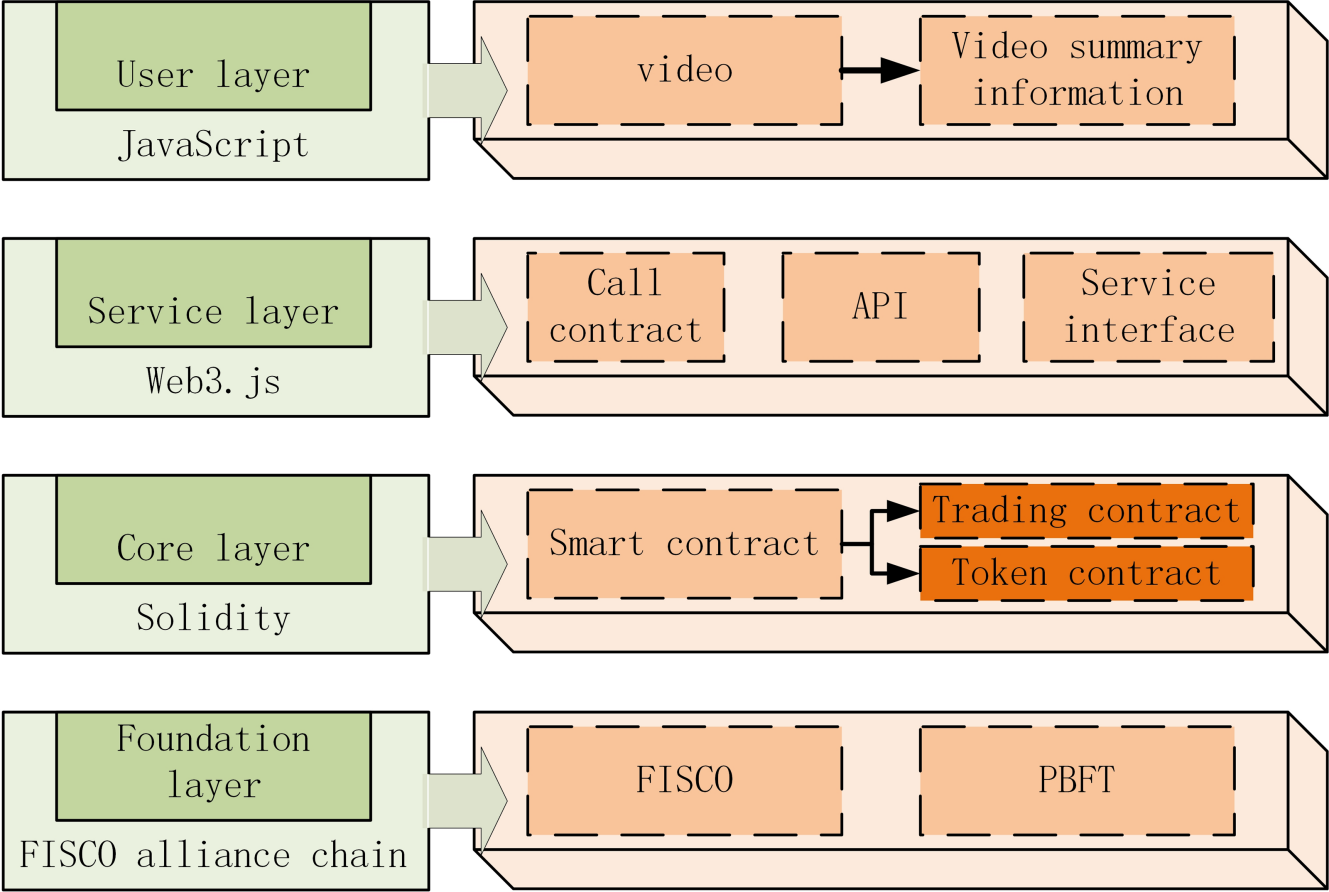
Transaction algorithm is divided.

The proposed algorithm is divided into three steps in the specific transaction. Firstly, among the videos waiting for transaction, the video that can be matched by *FISCO* alliance chain is priced. Secondly, match transaction execution. Finally, a smart contract is developed to supervise the execution of the transaction. The transaction algorithm is divided into base layer, core layer, service layer and user layer from bottom to top, as shown in Fig. 10.

**(a) Video intelligent pricing strategy**

In the video transaction algorithm based on *FISCO* alliance chain, some videos can be priced by member nodes independently. Meanwhile, in order to improve the transaction rate and execution efficiency, we must consider the short-term video transactions with the characteristics of “extremely fast output speed, extremely fast passing speed and extremely fast depreciation speed”. This paper innovatively proposes that the algorithm intelligently price the short videos, etc (hereinafter referred to as “pricing video”). “Massive, inexpensive, unified pricing” of video resources for intelligent pricing.

Suppose that there are *m* video media producers (sellers) in the member nodes of *FISCO* alliance chain, that is the seller *producer* ∈ {1, 2, …, *m*}; there are *n* video media buyers. These nodes as sellers can also become buyers when they have the intention to buy, so the maximum number of buyers may be *m+n*, that is *consumer* ∈ {1, 2, …, *m* + *n*}. The actual number of pricing videos sold by a member node in a transaction cycle is $vide{o}_{consumer}^{output}$, and the actual number of pricing videos purchased by a member node is $vide{o}_{consumer}^{input}$. The total number of pricing videos actually sold by all member nodes in a transaction cycle is *video*^*output*^, and the total number of pricing videos actually purchased is *video*^*input*^. Suppose that in a certain period, the average purchase price (initial value specified by the smart contract) of all member nodes for the pricing video is $pr{i}_{avg}^{in}$, and the average selling price (initial value specified by the smart contract) of all member nodes for the pricing video is $pr{i}_{avg}^{out}$, so the purchase price *pri*^*in*^ and sale price *pri*^*out*^ of the pricing video to be calculated by the smart pricing strategy in this period should be at the average price In the interval, as shown in Formula (21).

(21) \begin{eqnarray*}pr{i}^{in},pr{i}^{out}\in [pr{i}_{avg}^{in},pr{i}_{avg}^{out}]\end{eqnarray*}

In this paper, two different situations are considered to calculate the price of the pricing video:

**(1)** ***video***^***output***^ ≥ ***video***^***input***^

The total number of pricing videos actually sold by all member nodes in a transaction cycle is greater than the total number of pricing videos actually purchased, which means that the number of pricing videos is sufficient. In order to ensure the normal sale of pricing videos, the pricing strategy should be inclined to low price, that is, the sum of the total amount of selling pricing videos and the total amount of purchasing pricing videos should be equal to the total income of the seller, as shown in Formula (22).

(22) \begin{eqnarray*}(vide{o}^{output}-vide{o}^{input})\cdot pr{i}_{avg}^{in}+vide{o}^{input}\cdot pr{i}^{out}=vide{o}^{output}\cdot pr{i}^{in}\end{eqnarray*}

In the pricing strategy proposed in this paper, the buyers and sellers get the same additional income as those from the *FISCO* alliance chain exchange, as shown in Formula (23).

(23) \begin{eqnarray*}vide{o}^{input}\cdot (pr{i}_{avg}^{out}-pr{i}^{out})=vide{o}^{output}\cdot (pr{i}^{in}-pr{i}_{avg}^{in})\end{eqnarray*}

According to the above Formula, the purchase price *pri*^*in*^ and the sale price *pri*^*out*^ can be calculated, as shown in Formula (24) and Formula (25).

(24) \begin{eqnarray*}& pr{i}^{in}=pr{i}_{avg}^{in}+vide{o}^{input}\cdot (pr{i}_{avg}^{out}-pr{i}_{avg}^{in})/2vide{o}^{output}\end{eqnarray*}

(25) \begin{eqnarray*}& pr{i}^{out}=(pr{i}_{avg}^{in}+pr{i}_{avg}^{out})/2\end{eqnarray*}

**(2)** ***video***^***output***^ < ***video***^***input***^

The total number of pricing videos actually sold by all member nodes in a transaction cycle is less than the total number of pricing videos actually purchased, which means that the number of pricing videos is insufficient. In order to ensure the normal sale of pricing videos, the pricing strategy should be inclined to high price, that is, the sum of the amount of extra expenses and the total revenue of the seller is the total expenditure of the buyer, as shown in Formula (26).

(26) \begin{eqnarray*}(vide{o}^{input}-vide{o}^{output})\cdot pr{i}_{avg}^{out}+vide{o}^{output}\cdot pr{i}^{in}=vide{o}^{input}\cdot pr{i}^{out}\end{eqnarray*}

According to the above Formula, the purchase price *pri*^*in*^ and the sale price *pri*^*out*^ can be calculated, as shown in Formula (27) and Formula (28).

(27) \begin{eqnarray*}& pr{i}^{in}=(pr{i}_{avg}^{in}+pr{i}_{avg}^{out})/2\end{eqnarray*}

(28) \begin{eqnarray*}& pr{i}^{out}=pr{i}_{avg}^{out}-vide{o}^{output}\cdot (pr{i}_{avg}^{out}-pr{i}_{avg}^{in})/2vide{o}^{input}\end{eqnarray*}

**(b) Video transaction algorithm**

**(1) Token and incentive mechanism of** ***FISCO*** **alliance chain**

The algorithm will set a certain number of initial tokens for each member node. The initial token is directly transferred to the trading smart contract account through the process specified in the smart contract, which is called the minimum admission fee. The role of this rule is to prevent the remaining tokens from being insufficient to pay for the actual purchase of video in the previous period in the transaction settlement stage. After the transfer of the minimum admission fee is successful, the smart contract will create an admission fee data structure for the member node, as shown in Algorithm 1.

**Table:** 

**Algorithm 1: Admission data structure**
Struct Deposit{
address EthereumAddress;//Ethereum account address of member node
uint256 DepositNum;//Member node margin quantity
}

After the data amount of member node reaches the amount specified in the smart contract, it enters the pre transaction stage. In this stage, the seller of the pricing video submits the forecast sales volume of the next cycle through the video volume function, and the buyer pays in advance through the prepaid pricing video transaction fee. Before the prepaid transaction fee is used up, the buyer can continue to purchase the pricing video.

(2) **Settlement strategy**

The video sold by the member node, or the data generated by the purchase of video, is uploaded to the *FISCO* alliance chain network through the actual transaction function. The data package sent when uploading contains the summary information of the sale/purchase video, the user’s Ethereum account address, and the digital signature generated by using the user’s private key. The function of digital signature is to verify whether the packet is real and valid, as shown in Algorithm 2.

**Table:** 

**Algorithm 2: Pricing video transaction algorithm**
**Input**: $vide{o}_{consumer}^{output},vide{o}_{consumer}^{input},pr{i}_{avg}^{in},pr{i}_{avg}^{out}$
**Output**: The income of the seller *producer* is *profit*_*producer*_, and the amount paid by the buyer is *pay*_*consumer*_.
initialization:*video*^*input*^ = 0, *video*^*output*^
$vide{o}^{output}={\mathop{\sum }\nolimits }_{i=1}^{m}vide{o}_{i}^{output}$
$vide{o}^{input}={\mathop{\sum }\nolimits }_{j=1}^{m+n}vide{o}_{j}^{input}$
if*video*^*output*^ ≥ *video*^*input*^
According to Formula(24)and Formula(25), the purchase price*pri*^*in*^and the sale price*pri*^*out*^are calculated
else
According to Formula(27)and Formula(28), the purchase price*pri*^*in*^and the sale price*pri*^*out*^are calculated
end if
for*i* = 0;i<=m;i++ do
$profi{t}_{producer}=pr{i}^{out}\cdot vide{o}_{i}^{output}$
*end for*
for*j* = 0;j<=m+n;j++ do
$pa{y}_{consumer}=pr{i}^{in}\cdot vide{o}_{j}^{input}$
*end for*


**(c) Strategy of smart contract making**

The algorithm proposed in this paper is divided into two modules in the aspect of intelligent contract making. *FISCO* alliance chain token contract and transaction contract. Because the transaction of member nodes in *FISCO* alliance chain is cyclical and corresponding to real world currency. Therefore, in order to facilitate the settlement between financial institutions, the *FISCO* alliance chain token should have stable value. The consensus mechanism of *POA* does not have the feature of block reward, which makes it necessary for financial institutions to issue tokens. The token set in this paper is based on *ERC233* standard, as shown in Algorithm 3.

**Table:** 

**Algorithm 3: Token contract function**
function totalSupply() constant returns (uint256 totalSupply)
function name() constant returns (string_name)
function symbol() constant returns (byte32_symbol)
function decimals() constant returns (uint8_decimals)
function balanceOf(address_owner) constant returns (uint256 balance)
function transfer(address_to,uint_value) returns (bool)
function transfer(address_to,uint_value,bytes_data) returns (bool)
function tokenFallback(address_from,uint_value,byets_data)


Where *balanceOf(address_owner)* function can query the number of *FISCO* alliance chain representatives, *transfer(address_to,uint_value)* function can transfer the *FISCO* federation chain token to another account address.

**Figure 11:**
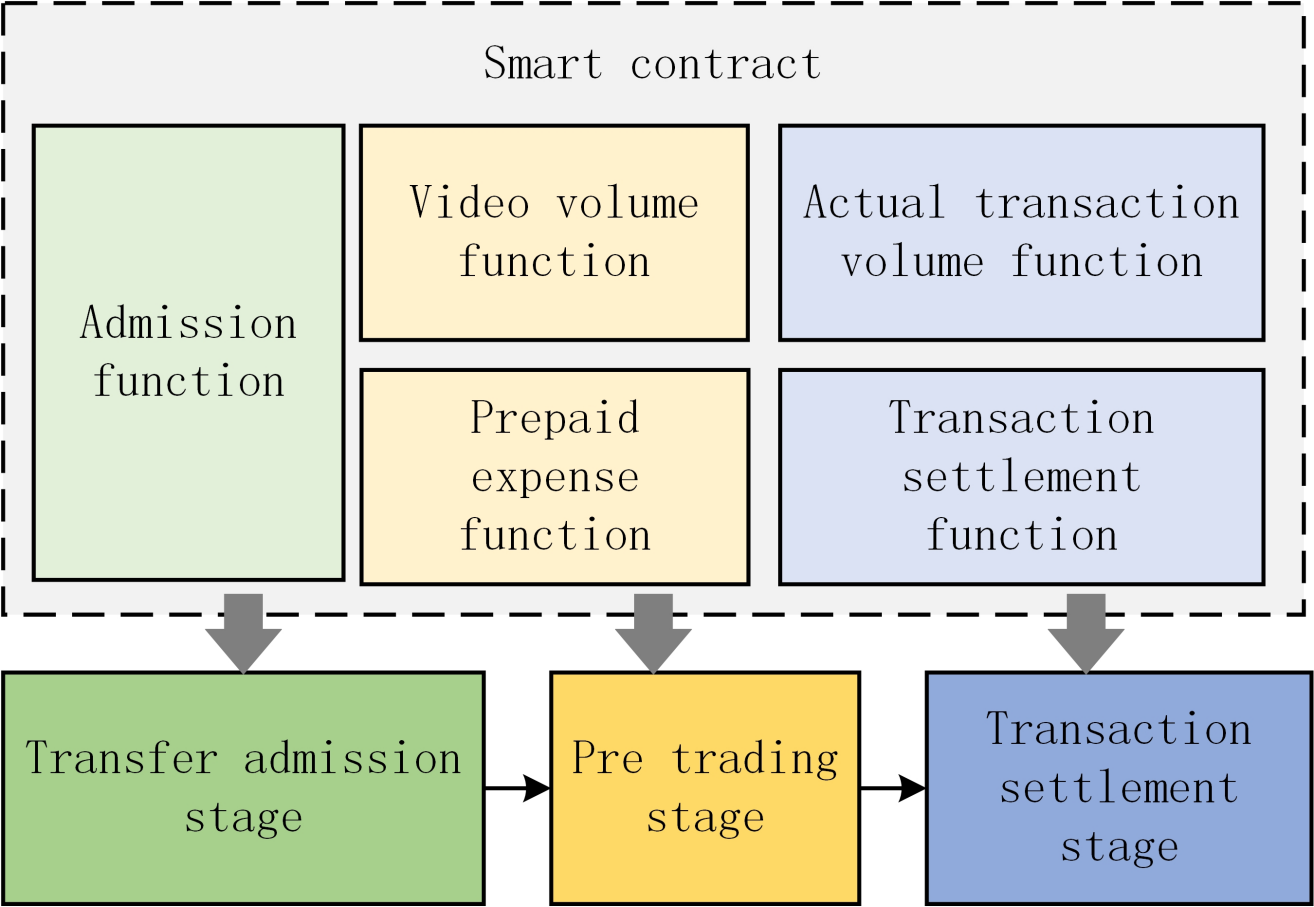
Actual transaction volume function and transaction settlement function.

The transaction contract is issued by the member nodes of the *FISCO* alliance chain specified by the algorithm, and the video transactions based on the *FISCO* alliance chain are realized through the transaction contract, which mainly includes five core functions: admission function, video volume function, prepayment function, actual transaction volume function and transaction settlement function, as shown in Fig. 11.

### Experiment

**Figure 12:**
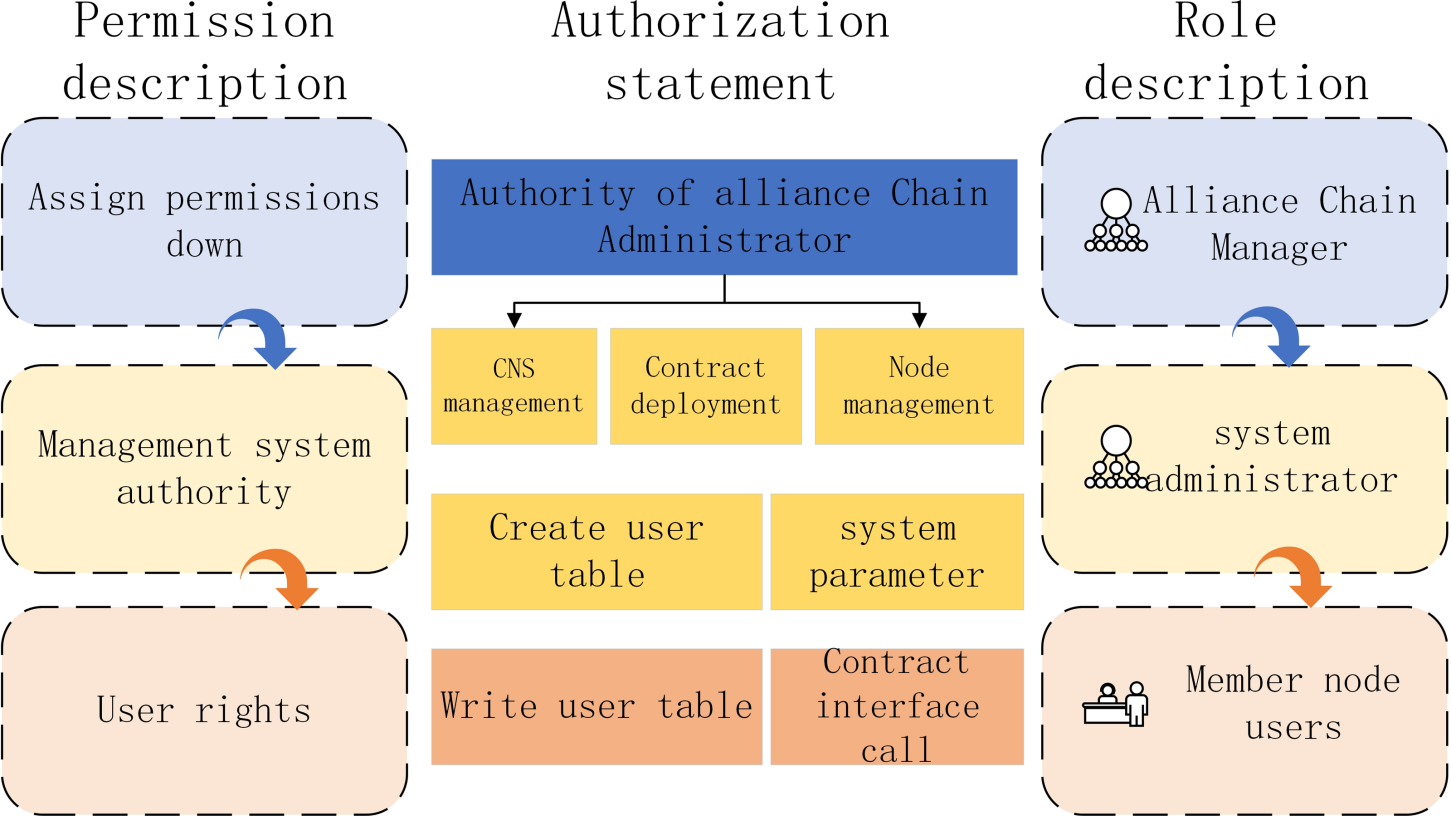
*FISCO* alliance chain member nodes.

In the algorithm proposed in this paper, *FISCO* alliance chain innovatively proposes a distributed storage permission control scheme in order to control the permissions of alliance chain more flexibly. Based on external account access, the algorithm controls the rights of contract deployment and read-write table operation. The transaction process can be monitored and traced. In the experiment, *FISCO* alliance chain member nodes include alliance chain administrator, system administrator, member node user and smart contract, as shown in Fig. 12.

In the experiment, after the member nodes of *FISCO* alliance chain initiate the transaction request through the client, the member nodes get the data about the transaction. Through the data to obtain the external account, obtain the table to be operated and the operation table.

The digital ID card contains public information and encrypted information. The public information includes video ID, storage location, owner, video summary and generation time; The encrypted information includes the video source code obtained in the previous step, watermark information coding and video fingerprint information coding.

After getting this digital ID card, all videos involved in the transaction method are uniquely identified by this digital ID card.

**Figure 13:**
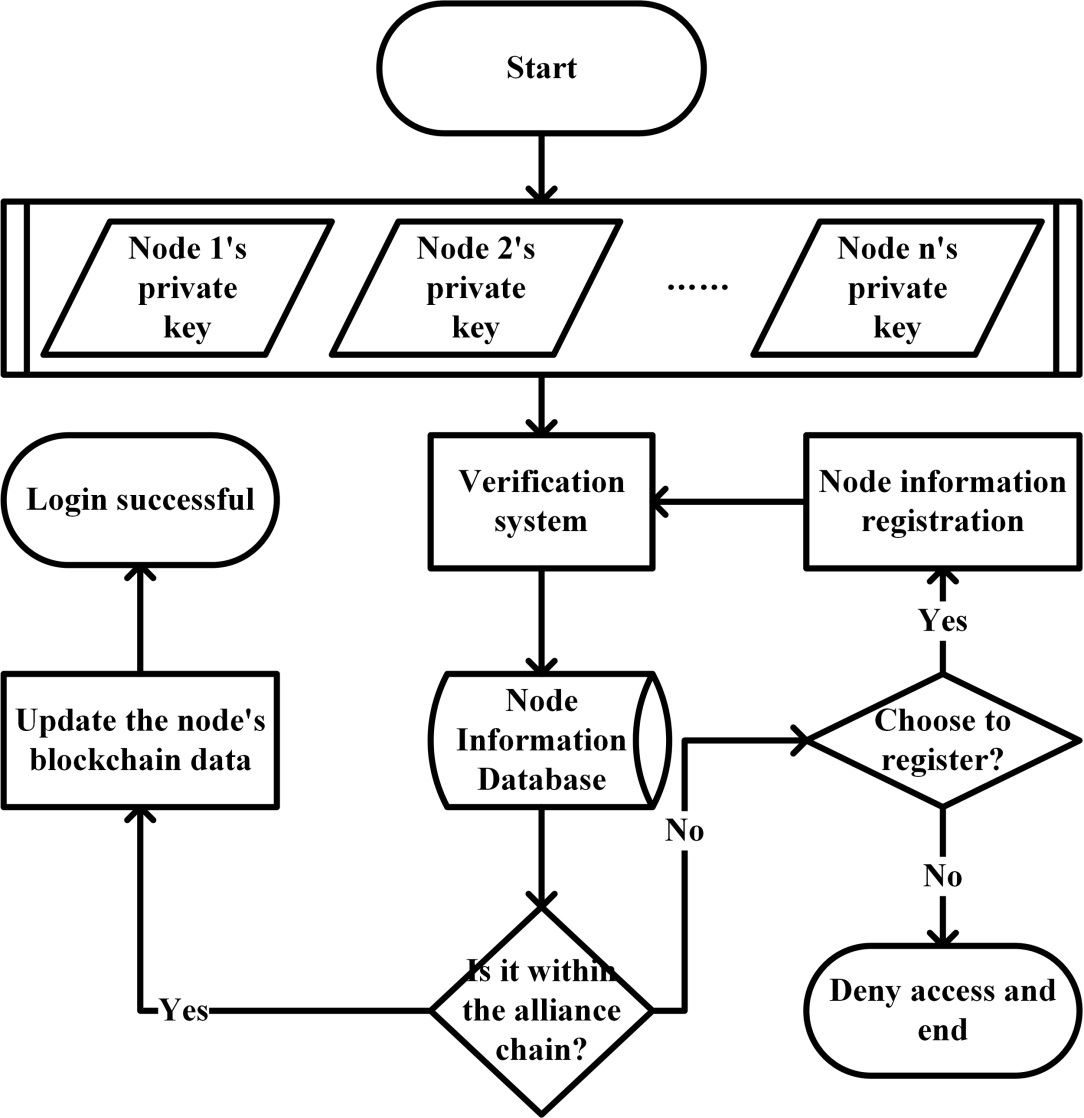
Identity authentication of the parent chain member node.

If the node chooses to register, it will submit its basic information and wait for the existing member nodes to confirm in turn, and then return to log in again after the confirmation. If you do not choose to register, the system denies the node’s access request. The identity authentication of the parent chain member node is shown in Fig. 13.

**Figure 14:**
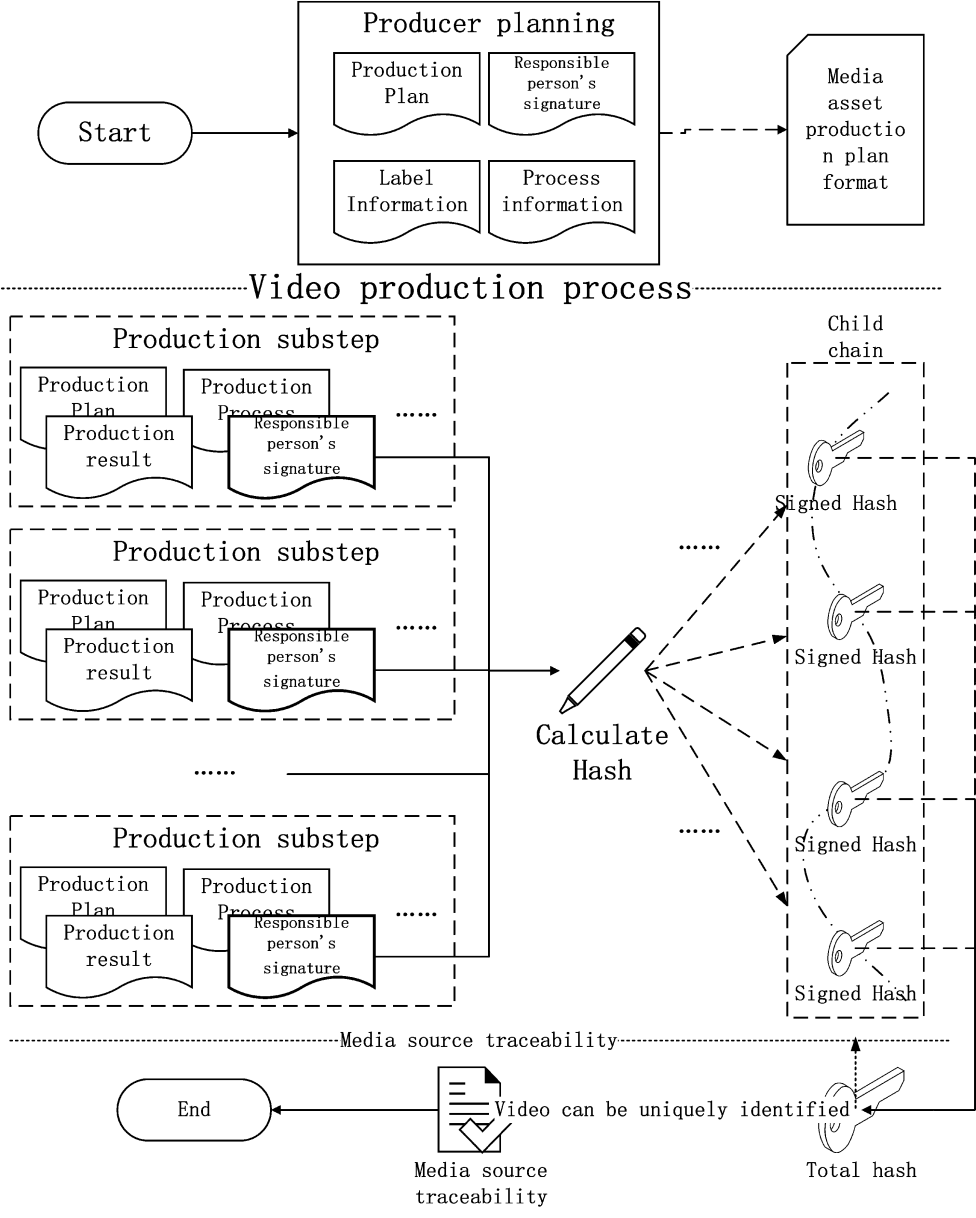
The calculation process of the video traceability source code.

The smart contract will integrate the process information and the signature of the person in charge of this step to perform a hash operation, and obtain the hash code of this step as label information and store it in the private area. In the blockchain, the current progress of the video work is then transferred to the video improvement team. the calculation process of the video traceability source code is shown in Fig. 14.

**Figure 15:**
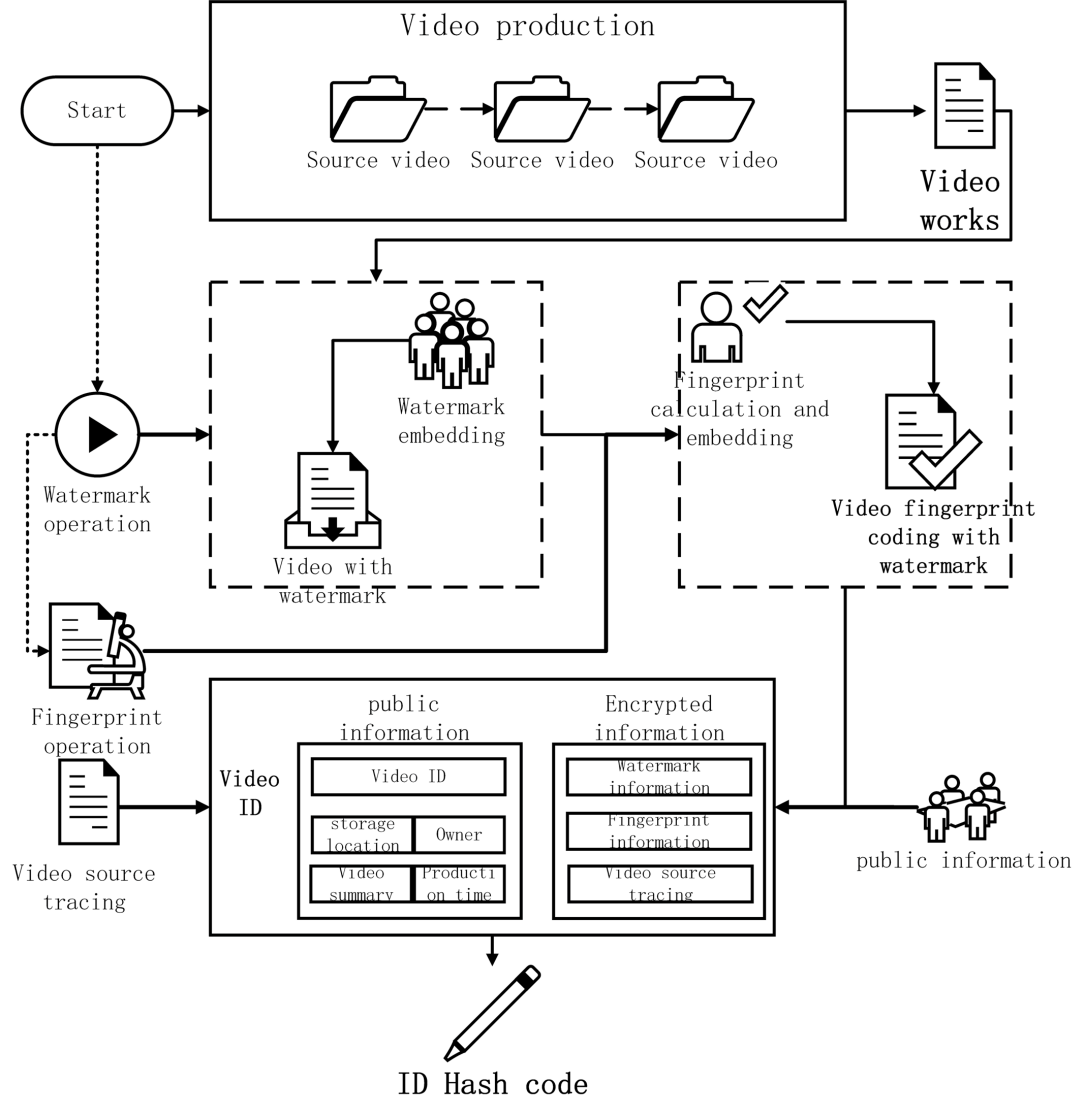
The calculation process of the video watermark code.

Establish a video ID card, which contains two sets of information: public information-video ID, storage location, party of the video, brief introduction of the video and the time the video was generated; encrypted information-video watermark code, video fingerprint code and video Trace the source code. Calculate the HID code of the video ID card for all the information of the video ID card. This Hash code can represent the operation of the on-chain storage and transaction information on the video. the calculation process of the video watermark code, fingerprint code and ID card is shown in Fig. 15.

### Experiment background, running environment and configuration file

*FISCO* alliance chain and improved trusted computing provide a trusted, traceable and provable transaction environment for video transaction, and use hierarchical intelligent contract structure to manage the transaction. Among them, the factory contract is agreed by all parties of the member node in advance, which stores the effective conditions of the transaction and manages the generation of the transaction; the transaction contract is generated by the factory contract, which stores the unique identification serial number (ID), *Hash* value and signature value of all parties, that is, each transaction has a contract. The interaction between factory contract and trade contract can make the system more scalable. The main business process of the experiment is to collect and analyze the public key information of all the member nodes in the *FISCO* alliance chain and deploy the factory contract, then in the process of transaction, the video seller sends the signed transaction information and broadcast the uplink request, the member nodes receive the uplink request, take out the transaction information and confirm and sign, and finally complete the uplink operation of the transaction information.

The experiment will be based on the Ubuntu 20.04 64bit system using the development tool *build_ chain.sh* Deploy a 4-node *FISCO* federation chain, download the console and update the public and private key information of the signature authority after the completion of the construction, and finally copy the node certificate.

This paper experimentally conFigures the role public key of the *FISCO* federation chain configuration file *applicationContext*, where *User* is the member node user and the corresponding private key file is *user.jks*. *Arbitrator* is the member node of transaction verification, and the corresponding private key file is *arbitrator.jks*. *Depositor* is the member node of transaction storage, and the corresponding private key file is *depositor.jks*.

### Description of data set and Experiment Kit

The video material used in the paper experiment is from the public data set HMDB51 (Kuehne et al., 2013), which contains 6849 videos from Youtube and Google. The paper experiment will randomly extract videos from the data set, extract video summary information, and then trade the summary information among the members of *FISCO* alliance chain.

In the experiment, the execution script is stored in *bin* folder, the toolkit configuration file is stored in *conf* folder, the transaction algorithm dependent package is stored in *lib* folder, and the smart contract is stored in *contracts* folder. Firstly, the environment of the toolkit is initialized, and the public–private key pair is updated according to the actual needs, in which the public key is stored in the configuration file in the form of *key-value*.

*FISCO* alliance chain provides intelligent contract interface development mode, which can operate the creation table, add, delete, modify and query data table in the database through intelligent contract. Moreover, intelligent contract needs to be compiled into *ABI* file and *BIN* file before it can be deployed to *FISCO* alliance chain. This file can be deployed and called by virtue of *JavaSDK*. *java* directory generated *org/FISCO/bcos/asset/contract/* package path directory, the directory contains *Asset.java* and *Tablejava* Two files, of which *Asset.java,* it’s a *Java* application call *Asset.sol* documents required for the contract. Thesis experiment in *build.gradle* Add a reference to *FISCO bcos Java SDK* under *dependencies* in the file and modify the *build.gradle.* The file introduces the spring framework.

The experiment configuration *SDK* certificate is consistent with the *Java SDK* configuration. In the experiment, there are initialization code, construction of contract class object and interface call in the calling of *FISCO* alliance chain *Java SDK*.

### Client instructions

The call of smart contract in the experimental client mainly includes initializing *WEB3J*, deploying contract object, loading deployed contract, creating transaction, sending transaction signature data, obtaining transaction details and verifying transaction correctness. The experimental process of this paper can be summarized as follows: the *FISCO* federation chain node communicates with the *FISCO* federation chain client node through the *Java-Wrapper* class generated by *WEB3J* and the *JsonRpc* call, and then the client returns the response request *JsonRpc* to the node.

**Figure 16:**
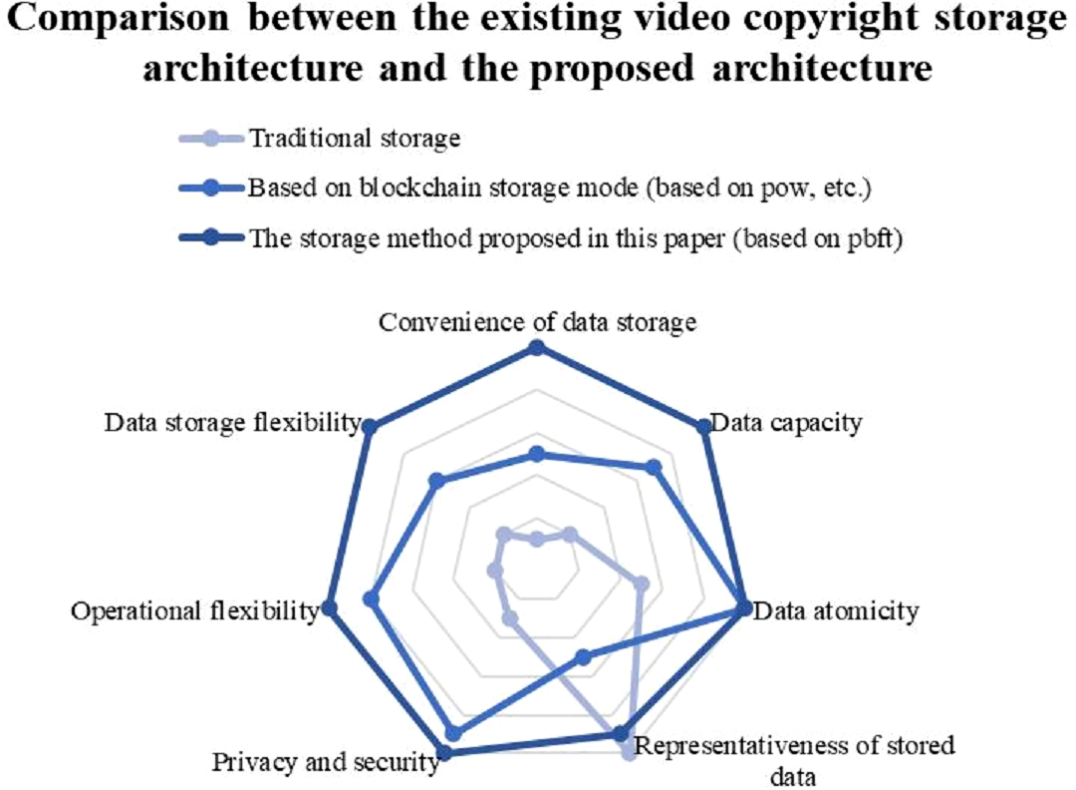
This paper compares the experimental results of [Traditional storage] and other two algorithms in six aspects, such as [Convention of data storage]. (1) The [Traditional storage] algorithm is better than the other two algorithms in [Representativeness of stored data]; (2) [Based on blockchain storage mode] algorithm has no advantage in [Representativeness of stored data], but it is superior to [Traditional storage] algorithm in other aspects; (3) The algorithm proposed in this paper is slightly behind the [Traditional storage] algorithm in terms of [Representativeness of stored data]. In any other aspect, the algorithm is far better than the other two.

Compared with the traditional video transaction algorithm, the video transaction algorithm considering FISCO alliance chain and improved trusted computing proposed in the paper is efficient and can make obvious progress in many aspects, In terms of [convention of data storage], [data capacity], [data storage flexibility], [operational flexibility], the algorithm proposed in this paper has advantages, which greatly improves the performance of pow algorithm and traditional storage, In the aspect of [data atomicity] [privacy and security], the algorithm proposed in this paper is not greatly improved, but also improved. In the aspect of representativeness of stored data, the algorithm proposed in this paper has no obvious improvement compared with the traditional storage, which is the problem to be studied in the next step, as shown in Fig. 16.

## Conclusions

This paper provides a new algorithm for video transaction. The experimental results show that the method performs well in robustness and efficiency. This paper first solves the problem that large video files cannot be traded on the chain by extracting video abstract information. Then, through the improved trusted computing, the non Byzantine attack in the transaction process is solved. The next research goal of this paper is how to store the large capacity video on the chain, and how to encrypt the transaction details.

##  Supplemental Information

10.7717/peerj-cs.594/supp-1 Supplemental Information 1 *WEB3J* initialization core code

10.7717/peerj-cs.594/supp-2 Supplemental Information 2 Contract deployment core code

10.7717/peerj-cs.594/supp-3 Supplemental Information 3 Call object core code

10.7717/peerj-cs.594/supp-4 Supplemental Information 4 Return the response core code

10.7717/peerj-cs.594/supp-5 Supplemental Information 5 Signature core code

10.7717/peerj-cs.594/supp-6 Supplemental Information 6 Get transaction details core code

10.7717/peerj-cs.594/supp-7 Supplemental Information 7 Transaction correctness verification core code

## Additional Information and Declarations

### Data Availability

The following information was supplied regarding data availability: The data is available at HMDB51:
http://serre-lab.clps.brown.edu/resource/hmdb-a-large-human-motion-database/#Downloads
The program code is available at GitHub: https://github.com/hueihan/Action_Recognition.
